# The humanised *CYP2C19* transgenic mouse exhibits cerebellar atrophy and movement impairment reminiscent of ataxia

**DOI:** 10.1111/nan.12867

**Published:** 2023-01-17

**Authors:** Filip Milosavljević, Irene Brusini, Andrea Atanasov, Marina Manojlović, Marija Vučić, Zorana Oreščanin‐Dušić, Jelena Brkljačić, Čedo Miljević, Aleksandra Nikolić‐Kokić, Duško Blagojević, Chunliang Wang, Peter Damberg, Vesna Pešić, Rachel F. Tyndale, Magnus Ingelman‐Sundberg, Marin M. Jukić

**Affiliations:** ^1^ Department of Physiology, Faculty of Pharmacy University of Belgrade Belgrade Serbia; ^2^ Department of Biomedical Engineering and Health Systems KTH Royal Institute of Technology Huddinge Sweden; ^3^ Department of Neurobiology, Care Sciences and Society Karolinska Institute Huddinge Sweden; ^4^ Institute for Biological Research "Siniša Stanković" – National Institute of the Republic of Serbia University of Belgrade Belgrade Serbia; ^5^ Department of Psychiatry, Faculty of Medicine University of Belgrade Belgrade Serbia; ^6^ Institute for Mental Health Belgrade Serbia; ^7^ Karolinska Experimental Research and Imaging Center Karolinska University Hospital Solna Sweden; ^8^ Campbell Family Mental Health Research Institute Centre for Addiction and Mental Health Toronto Ontario Canada; ^9^ Department of Psychiatry University of Toronto Toronto Ontario Canada; ^10^ Department of Pharmacology and Toxicology University of Toronto Toronto Ontario Canada; ^11^ Section of Pharmacogenetics, Department of Physiology and Pharmacology Karolinska Institutet Stockholm Sweden

**Keywords:** animal models, cerebellar ataxia, cerebellum, cytochrome P‐450 Cyp2c19, movement disorders, neuroimaging, transgenic mice

## Abstract

**Aims:**

*CYP2C19* transgenic mouse expresses the human *CYP2C19* gene in the liver and developing brain, and it exhibits altered neurodevelopment associated with impairments in emotionality and locomotion. Because the validation of new animal models is essential for the understanding of the aetiology and pathophysiology of movement disorders, the objective was to characterise motoric phenotype in *CYP2C19* transgenic mice and to investigate its validity as a new animal model of ataxia.

**Methods:**

The rotarod, paw‐print and beam‐walking tests were utilised to characterise the motoric phenotype. The volumes of 20 brain regions in *CYP2C19* transgenic and wild‐type mice were quantified by 9.4T gadolinium‐enhanced *post‐mortem* structural neuroimaging. Antioxidative enzymatic activity was quantified biochemically. Dopaminergic alterations were characterised by chromatographic quantification of concentrations of dopamine and its metabolites and by subsequent immunohistochemical analyses. The beam‐walking test was repeated after the treatment with dopamine receptor antagonists ecopipam and raclopride.

**Results:**

*CYP2C19* transgenic mice exhibit abnormal, unilateral ataxia‐like gait, clasping reflex and 5.6‐fold more paw‐slips in the beam‐walking test; the motoric phenotype was more pronounced in youth. Transgenic mice exhibited a profound reduction of 12% in cerebellar volume and a moderate reduction of 4% in hippocampal volume; both regions exhibited an increased antioxidative enzyme activity. *CYP2C19* mice were hyperdopaminergic; however, the motoric impairment was not ameliorated by dopamine receptor antagonists, and there was no alteration in the number of midbrain dopaminergic neurons in *CYP2C19* mice.

**Conclusions:**

Humanised *CYP2C19* transgenic mice exhibit altered gait and functional motoric impairments; this phenotype is likely caused by an aberrant cerebellar development.

## Graphical abstract

Transgenic mice, carriers of the human CYP2C19 gene, exhibit mild gait disturbance that is bilateral in young animals and unilateral in adults. They also exhibited performance impairment in the beam‐walking test and a reduction of 12% in the volume of cerebellum measured with ex vivo 9.4T gadolinium‐enhanced neuroimaging but showed no abnormalities in rotarod or footprint tests. This animal model exhibits an ataxia‐like motoric phenotype and can provide insight into the aetiology and pathophysiology of cerebellar ataxia.

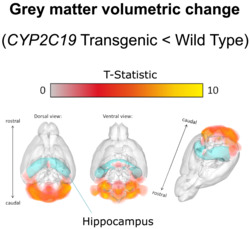

**Key points**

- *CYP2C19* humanised transgenic mice show mild motoric disturbance.
- Altered gait is bilateral in young *CYP2C19* mice but becomes unilateral in adulthood.
- Cerebellar volume is reduced by 12% in *CYP2C19* mice compared with controls.
- *CYP2C19* mice may be a useful model for the research of cerebellar ataxia.

## INTRODUCTION

Cerebellar ataxia is an umbrella clinical term used for the group of severe illnesses caused by various hereditary and/or acquired factors [1]. The clinical phenotype is heterogeneous among different subtypes of cerebellar ataxia; the most common symptoms include unstable gait, lack of balance, blurred vision, slurred speech and loss of fine dexterity [1]. Despite the major negative impact on the quality of life in affected individuals, the aetiology and pathophysiology of cerebellar ataxia are not completely understood, and effective treatment strategies for many forms of ataxia are sparse [2, 3]. Several animal models of cerebellar ataxia have been developed to clarify molecular and cellular mechanisms causing cerebellar dysfunction. Specifically, transgenic mice that carry gene variants found in human hereditary forms of ataxia and animals selectively bred for their ataxia‐like phenotype are frequently used [4]. These animal models have previously been very useful for the investigation of cerebellar function and ataxic pathophysiology [2]; consequently, the characterisation of novel animal models of ataxia is expected to further expand this knowledge.

*CYP2C19* humanised transgenic mouse is an animal model that can be useful in the research of cerebellar function and ataxia. This mouse carries human *CYP2C19* and *CYP2C18* genes, which do not possess orthologous mouse variants. Whereas the *CYP2C18* gene is not translated into protein in this animal model, the *CYP2C19* gene encodes the CYP2C19 human liver enzyme, which is also expressed in the human fetal brain, suggesting that the enzyme may affect brain development [5, 6]. The exact mechanism behind the influence of the CYP2C19 enzyme on neural development has not been directly determined; however, it has been hypothesised that the CYP2C19 enzyme catalyses endogenous substances in the fetal brain, which subsequently affect brain development [5, 7]. Known endogenous substrates of CYP2C19 include polyunsaturated fatty acids, sex hormones and endocannabinoids, all of which are important for the various aspects of neuronal development [5, 7]. The *CYP2C19* transgenic mouse is a very good tool to study the role of *CYP2C19* in the developing brain in vivo, because this mutant exhibits almost identical CYP2C19 enzyme expression pattern to humans, including the expression in the brain‐restricted development phase and abundant CYP2C19 enzyme concentrations in adult liver, comparable with human levels [8]. Previous research on the *CYP2C19* mice revealed their complex emotional phenotype that includes increased susceptibility to stress [6], elevated depression‐like behaviour [5], increased serotonergic turnover and decreased whole‐brain and hippocampal volumes [6]; in addition, alterations in motoric functions have also been observed but not systematically investigated [6]. To our knowledge, the only study testing the association between the *CYP2C19* genotype and any movement disorder is the study of Alonso‐Navarro et al., [9] which showed a significantly higher frequency of CYP2C19 intermediate metabolizer status in the cohort of patients with essential tremor compared with healthy controls. Even though there is still no obvious link between *CYP2C19* and ataxia, unique features of an animal model expressing the human *CYP2C19* gene can be a useful addition to the preclinical research of cerebellar function and dysfunction.

The initial aim of this study was an in‐depth characterisation of the motoric phenotype seen in *CYP2C19* humanised transgenic mice, whereas the subsequent aim was to examine its potential validity as an animal model for cerebellar ataxia. The primary hypothesis was as follows: (1) Motoric phenotype in *CYP2C19* transgenic mice resembles human ataxia and leads to impaired performance in locomotor tests. Secondary hypotheses were driven by preliminary data, in particular, (2) *CYP2C19* mice exhibit structural abnormalities in the brain regions associated with locomotion; (3) oxidative stress and/or neuromelanin accumulation are involved in the structural brain alterations; (4) structural and functional alterations of dopaminergic system are involved in the observed motoric phenotype.

## MATERIALS AND METHODS

### Laboratory animals

The *CYP2C19* transgenic mice were kept on the C57Bl/6JOlaHsd genetic background, and they are hemizygous carriers of the insert, which contains 12 copies of human *CYP2C19* and *CYP2C18* genes [7]. Animals were housed in groups of 3–5 animals per cage. Housing conditions included a 12 h light/dark cycle, controlled room temperature (22 ± 1°C), humidity (40–70%) and illumination; and ad libitum access to water and pelleted food. Mice of both sexes were included, and they were divided into two test groups, based on their genotype: *CYP2C19* transgenic mice and their *wild‐type* littermates. The body weight of animals was measured every day in the postnatal weeks 3–9. Investigators conducting the experiments were blinded to the animals' genotypes whenever this was possible. General signs of animal well‐being were checked at least twice a week, and if signs of severe distress were observed, such animals were euthanized to reduce their suffering. Animals showing abnormal behaviour such as repetitive movements or extreme agitation during handling were also excluded from the subsequent statistical analysis because this hinted at developmental disturbances caused by random factors in these animals. Most of the experiments were performed in mice of standard adult age of 3–6 postnatal months. There were two exceptions: gait analysis in young animals and structural analysis of the midbrain dopaminergic nuclei in 15 months old mice. All experiments were performed according to the permit of the Ethical Committee on Animal Experimentation of the University of Belgrade – Faculty of Pharmacy, Serbia (permit number 23‐07‐00933/2019‐05 to MMJ). The study was conducted and reported in accordance with ARRIVE 2.0 guidelines [10].

### Motor tests

Mouse gait was analysed with a footprint test [11] in adult mice at one time point (*CYP2C19* transgenic *n* = 15, *wild‐type n* = 15) and in young animals at four time points (*CYP2C19* transgenic *n* = 27, *wild‐type n* = 26) once a week in the postnatal weeks 5–8. Young animals were included as it was observed during animals' handling that gait changes during animals' maturation. Measured parameters included stride lengths for every paw, the width of the front and hind base and the overlap of the steps [11]. Next, the maximal height of hind paw elevation while walking was derived from the video footage of the footprint test. The validity of this measurement was ensured by the fixed placement of the camera during every run, exactly 18.5 cm from the apparatus, at a fixed height from the ground and a fixed angle compared with the apparatus. Also, only steps captured at the centre of the frame were considered for the analysis, and all runs were analysed by the same observer to avoid bias. Change in any of the parameters in *CYP2C19* transgenic animals compared with *wild‐types* would indicate the presence of gait disturbance. Mice were also visually screened for a pathological clasping reflex, which is present in numerous motorically impaired rodent strains [12].

Motoric function was quantified by the rotarod (*CYP2C19* transgenic *n* = 52, *wild‐type n* = 45, 3–5 months old animals) and beam‐walking (BW) tests (*CYP2C19* transgenic *n* = 48, *wild‐type n* = 37, 3–5 months old animals). An accelerating protocol was used in the rotarod test, and the latency to fall was measured [13]. An average of the three longest runs was used as a readout of motoric performance, because the motorically impaired mice tend to fall sooner than the non‐impaired animals [13]. In the BW test, a rectangular 8 mm wide beam was used. Because trained animals tend to cross the beam as fast as they possibly can, beam‐crossing time is prolonged mainly due to the presence of motoric disturbances [14]. Also, animals with poor motoric coordination tend to exhibit more paw slips during the beam‐crossing [14]. Hence, the performance in the BW test was measured as the average of the three shortest beam‐crossing times and as the average of the three smallest measured numbers of paw slips per run.

### Gd‐enhanced neuroimaging

To assess the structural integrity of various brain regions associated with motoric function, brains of 6 months old *wild‐types (n = 30)* and *CYP2C19* transgenic mice (*n* = 29) were investigated with gadolinium (Gd) enhanced *post‐mortem* neuroimaging. Additionally, volumes of non‐motoric regions were analysed either to replicate the previous finding of hippocampal atrophy [6] or for exploratory purposes. The samples were prepared for imaging through transcardial formaldehyde perfusion, supplemented with the magnetic resonance imaging (MRI) contrast agent gadoteridol (ProHance, Bracco Diagnostic Inc., NJ, USA) [15]. Scanning was performed using a 9.4 T horizontal bore MRI scanner (Varian, Yarnton, UK) equipped with a millipede coil with an inner diameter of 30 mm. The samples were scanned using a multi‐gradient echo 3D with the following parameters: matrix size 1024 × 512 × 512, field‐of‐view 51.2 × 25.6 × 25.6 mm^3^, recovery time 30 ms, time to echo 2.82 ms, flip angle 35°. Two echoes separated by 4.96 ms were acquired, and 512 dummy excitations to establish a steady state were used. Obtained data were segmented automatically based on the 3D Waxholm Space 2012 mouse brain atlas [16, 17] into 39 brain regions, but 19 regions such as ventricles, inner ear, medulla oblongata, various nerves and white matter structures were not analysed due to proneness of substantial variability induced by tissue processing. Volumes of each brain region were quantified by multiplying the number of voxels the region comprises with the volume of individual voxel (50 μm voxel edge, 12.5 × 10^−5^ mm^3^), whereas the total brain volume was calculated as the sum of volumes of all 39 brain regions.

Subsequently, between‐group differences in local grey matter (GM) volume were further investigated by performing voxel‐based morphometry (VBM). We employed FMRIB Software Library voxel‐based morphometry (FSL‐VBM) [18], an optimised VBM protocol [19] carried out with FSL tools [20]. Of note, the protocol was slightly modified to be adapted to the present mouse brain (see Supporting Information) images and to compensate for the difference between human and mouse brains. Local differences between groups were investigated by calculating voxel‐wise *F* statistics for genotype, with genotype and sex as factors.

### Antioxidative enzyme concentration and activity

Antioxidative enzyme status was determined in the brain tissues of 6 months old *wild‐types* (*n* = 32) and *CYP2C19* transgenic mice (*n* = 30) in the regions with the most pronounced structural change observed via neuroimaging in *CYP2C19* mice. This was done to explore the involvement of oxidative stress mechanisms in the atrophy observed in these regions. Expression and activities of four antioxidative enzymes: superoxide dismutase (SOD), catalase (CAT), glutathione peroxidase (GPx) and glutathione reductase (GR) were measured in the mouse cerebella, hippocampi and whole hemispheres. All enzyme activities were determined biochemically and expressed as units (U) per mg of protein, whereas enzyme expression levels were determined with the Western blot method and presented in arbitrary units.

### Dopamine level determination and dopamine receptor antagonist treatment

The concentration of dopamine and its two major metabolites, 3,4‐dihydroxyphenylacetic acid (DOPAC) and homovanillic acid (HVA), was determined with High performance liquid chromatography ‐ tandem mass spectrometry (HPLC‐MS/MS) method in the brain hemispheres, hippocampi and cerebella of adult 3 months old mice (*CYP2C19* transgenic *n* = 11, *wild‐type n* = 13) harvested after the decapitation as previously described [21]. Of note, this was the only experiment that included only males as the females had to be spared for the breading and colony expanding purpose at the time this experiment was conducted. Also, dopamine, DOPAC and HVA levels were measured in the whole brain samples of embryos at E18.5 day (*CYP2C19* transgenic *n* = 13, *wild‐type n* = 7).

Because the determination of dopamine concentration revealed hyperdopaminergism in adult *CYP2C19* mice, their dopaminergic system was characterised to assess its involvement in their motoric phenotype. First, the BW test (*CYP2C19* transgenic *n* = 47, *wild‐type n* = 42, 3–5 months old animals) was repeated after the treatment with the 0.1 mg/kg selective D1 antagonist ecopipam hydrobromide (SCH‐39166, Tocris Bioscience, UK) or the 0.25 mg/kg D2 antagonist raclopride (Tocris Bioscience, UK). If ecopipam administration was to reverse motoric impairment in *CYP2C19* mice observed in the BW test, this would indicate that excessive dopamine in their brains overactivated the D1 receptor in the striatum causing the impairment this way, and the same would be true for raclopride administration and D2 receptor involvement. Drugs were dissolved in a saline solution and administered in a single intraperitoneal injection 30 min before the BW test. Doses were selected to be sufficient to impact the performance of animals with hyperactivated dopaminergic systems but not to cause general sedation of the animals, as previously described in the literature [22].

### Immunohistochemistry and neuromelanin staining

Hyperdopaminergism observed in the adult *CYP2C19* mice most likely originates from the overactive midbrain dopaminergic neurons, as this is the biggest population of dopaminergic neurons in mammalian brains. This overactivation, depending on its intensity and duration, could cause excitotoxicity and dopaminergic neuron death in either adult life or the late life of *CYP2C19* mice. To assess this, we immunohistochemically stained slides of mouse brain sections to investigate the structures of mid‐brain dopaminergic nuclei in mice of different ages. Tyrosine hydroxylase positive (TH+) cells were counted in *substantia nigra* (SN) and *ventral tegmental area* (VTA) (staining validation presented in Figure 5C) and compared in nine transgenic/*wild‐type* littermate pairs; four pairs were adults (6 months old), and five pairs were at the old age (15 months old). Nine representative coronal slides were analysed and the neuron was considered TH + if the cytoplasm was stained and if the nucleus was visible.

Neuromelanin is a side‐product of catecholamine synthesis and is potentially involved in the pathogenesis of dopamine‐induced cell death in humans [23], and because *CYP2C19* mice are hyperdopaminergic, it is possible that neuromelanin aggregates in their midbrain dopaminergic neurons due to the higher‐than‐normal dopamine production. The presence of neuromelanin was assessed in the brain sections of 15 months old mice, stained with Fontana‐Masson melanin stain (ab150669, Abcam, UK) according to the manufacturer‐supplied protocol [24]. A histological sample of the human skin was used as a positive control.

### Statistical data interpretation

Data were analysed with SPSS statistics 20 software (IBM, USA). Genotype was the principle independent variable in all experiments. Determination of sample sizes and power analysis are presented in detail in the Supporting Information. In short, the rotarod, BW test (both preliminary and after pharmacological treatments), neuroimaging and oxidative stress analyses involved the same *n* = 99 cohort of animals. Originally, power analysis [25] estimated that *n* = 126 animals are needed to detect mild motoric disturbance (10% change) with 95% confidence, 80% power and 20% standard deviation known from the pilot test [25], but this number was reduced to *n* = 99 due to ethical concerns. Other tests utilised the smallest number of animals needed to yield meaningful results.

The normality of data distributions was assessed with the Shapiro–Wilk test; if the distribution was normal, all outliers were excluded based on the 2.2 interquartile range (IQR) rule [26]. If the experiment included covariates such as age or sex, one‐way analysis of covariance (ANCOVA) was performed; otherwise, Student's *t* test for independent samples was used. Exceptions were experiments that included drug treatments or multiple time points where two‐way mixed ANCOVA was performed and non‐normally distributed data where Mann Witney and Kruskal Wallis tests were used. All tests were two‐tailed with a *p* value < 0.05 as a measure of significance. False discovery rate (FDR) correction of *p* values for multiple comparisons was applied when multiple brain regions or multiple antioxidant enzyme activities were analysed. Differences between test groups were textually presented as the ratio‐of‐means with a 95% confidence interval (95% CI) for normally distributed data or as median and IQR for each group for non‐Gaussian data. For the VBM analysis, the threshold‐free cluster enhancement method was used to obtain corrected voxel‐wise *t*‐ and *F*‐statistics for genotype, with sex and genotype as factors. Experimental protocols were pre‐specified during the ethics committee permit acquirement process, whereas the footprint analysis was the only post hoc test. Detailed descriptions of all experimental procedures and statistical analyses are available in the Supporting Information.

## RESULTS

**FIGURE 1:**
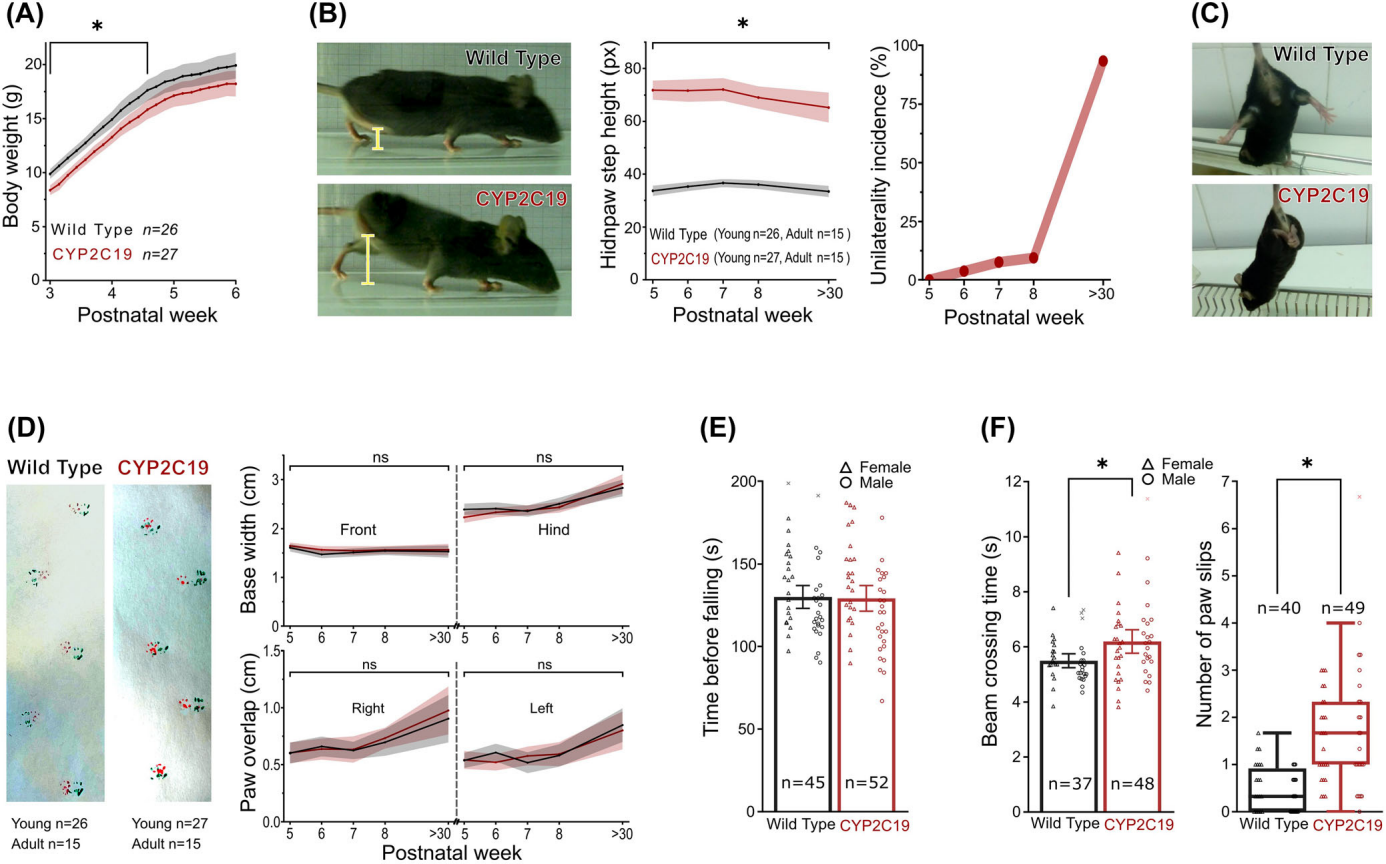
Motoric phenotype in *CYP2C19* transgenic mice. *CYP2C19* transgenic mice exhibited significantly slower weight gain during development (*F*_1,51_ = 6.29, *p* = 0.015) until postnatal day 31 when this difference stops being significant (A). *CYP2C19* transgenic mice experience abnormal hind paw elevation in youth (2.1‐fold [95% CI: 2.0, 2.2], *F*_1,51_ = 415, *p* < 0.0001) and in adulthood (1.9‐fold [95% CI: 1.8, 2.1], *F*_1,28_ = 129, *p* < 0.0001) (B) and clasping reflex (C). No abnormalities were found in *CYP2C19* transgenic mice in any of the parameters measured in the footprint test (D) and *rotarod* test (*F*_1,97_ = 0.008, *p* = 0.93) (E). *CYP2C19* transgenic mice performed worse than *wild‐types* in the beam‐walking test with longer (1.14‐fold, [95% CI: 1.06, 1.22], *F*_1,83_ = 10.9, *p* = 0.0014) beam‐crossing time and a greater number of paw slips (*CYP2C19* transgenic: 1.7 [interquartile range (IQR): 1.0, 2.3] vs *wild‐type* 0.33 [IQR: 0.0, 0.92], *U* = 298, *p* < 0.0001) (F). Data presented as means with whiskers/area as 95% CI or as Tukey box‐plot. *Statistical significance p < 0.05.

*CYP2C19* transgenic animals exhibited slower body growth (5–10% lower body weight) during the 3rd and 4th postnatal week, whereas after postnatal day 31, the differences in the body weight were no longer statistically significant (Figure 1A). The *CYP2C19* transgenic animals exhibited around a two‐fold increase in maximal hind paw elevation in both young and adult mice (Figure 1B), compared with *wild‐type* controls. However, while all young transgenic mice exhibited an increase in elevation of both hind paws, in the 5th postnatal week, they gradually started exhibiting unilateral phenotype until almost all (49 of 51) animals became unilaterally affected in adulthood (Table S5, Video S1). The number of adult *CYP2C19* transgenic mice with affected left vs right hind paws was similar. In addition, the pathological clasping reflex, which occurred after several seconds of struggle upon elevation from the firm surface, was observed in all tested *CYP2C19* transgenic animals (Figure 1C). Next, gait analysis did not show significant genotype‐specific changes in young and adult animals (Figure 1D). There was a trend towards the 7% narrower hind base in *CYP2C19* transgenic mice at the 5th postnatal week, but the effect was no longer significant after the correction for animals' sex. Several animals were excluded from the motoric tests because they were unmotivated to complete the tasks or the training; a similar number of exclusions occurred in transgenic and *wild‐type* mice. Animals excluded for this reason lost motivation gradually, indicating habituation to the test conditions.

To investigate the potential functional implications of the observed motoric phenotype in *CYP2C19* transgenic mice, the latency to fall was measured on the rotarod test, whereas the beam‐crossing time and number of paw slips were measured on the BW test. On the rotarod test, no significant motoric impairment was observed, as *CYP2C19* transgenic and *wild‐type* mice did not exhibit different latencies to fall (Figure 1E). However, impairment of motor function was observed on the BW test, as *CYP2C19* transgenic mice exhibited slightly, 1.14‐fold longer beam crossing times and profoundly 5.6‐fold increased numbers of paw slips, compared with *wild‐types* (Figure 1F). In summary, *CYP2C19* transgenic mice exhibit a visually pronounced motoric phenotype, which is in its true nature subtle and impacts motoric function only in challenging tasks.

**FIGURE 2:**
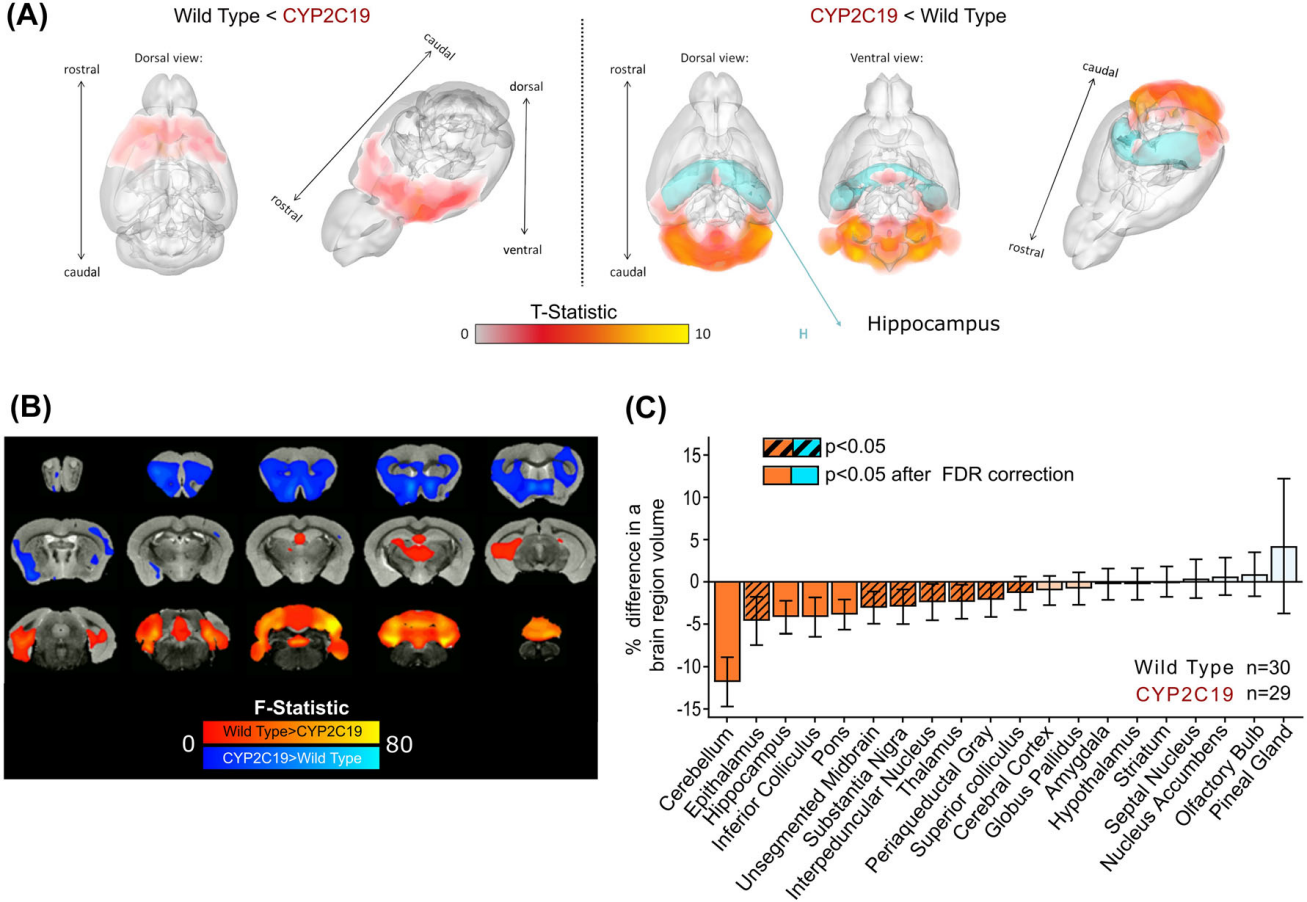
Structural changes in brains of *CYP2C19* transgenic mice measured with 9.4T magnetic resonance imaging (MRI) neuroimaging. Volumetric differences between *CYP2C19* transgenic mice and *wild‐types* analysed with voxel‐based morphometry are presented as (A) 3D rendering and (B) coronal cross‐sections. Panel (C) presents magnitudes of unadjusted volumetric changes in *CYP2C19* transgenic mice in 20 segmented regions of interest: cerebellar volume (−11.8% [95% CI: −14.7%, −9.0%], *F*_1,57_ = 69.1; *p* < 0.0001, *q* < 0.0001), hippocampus (−4.2% [95% CI: −6.1%, −2.3%], *F*_1,57_ = 18.4, *p* = 0.0001, *q* = 0.0027), pons (−3.9% [95% CI: −5.6%, −2.1%], *F*_1,57_ = 19.6, *p* < 0.0001, *q* = 0.0017), epithalamus (−4.6% [95% CI: −7.4%, −1.9%], *F*_1,57_ = 12.5*, p* = 0.0008, *q* = 0.030) and inferior colliculus (−4.2% [95% CI: −6.4%, −1.9%], *F*_1,57_ = 14.1, *p* = 0.0004, *q* = 0.015). *Bars and whiskers represent means and 95% CI.*

Next, potential neuroanatomical changes in *CYP2C19* transgenic mice were investigated throughout the brain with Gd‐enhanced ex vivo structural MRI neuroimaging. Significant, structural GM alterations identified through VBM are graphically represented in 3D (Figure 2A) and as a collection of representative coronal neuroimaging slides (Figure 2B). Because the total brain volume in the *CYP2C19* transgenic mice was significantly reduced by 3%, volumes of the 20 segmented and analysed regions of interest were not corrected for the total brain volume of respective animals. Significant volumetric changes in *CYP2C19* transgenic mice compared with *wild‐types* were observed in 10 out of 20 regions, out of which five remained significant after multiple comparisons correction. *CYP2C19* transgenic mice exhibited a profound decrease of 12% in the cerebellar volume and a moderate reduction of 4% in the hippocampal volume, compared with the corresponding volumes measured in *wild‐type* mice. Also, moderate volumetric decreases in pons, epithalamus and inferior colliculus were observed in *CYP2C19* transgenic mice, compared with *wild‐types* (Figure 2C). The effects of covariates on the measured outcomes were not pronounced and are presented in detail in the Supporting Information. In summary, *CYP2C19* transgenic mice exhibit a structural brain phenotype, which includes cerebellar and hippocampal volumetric decreases.

**FIGURE 3:**
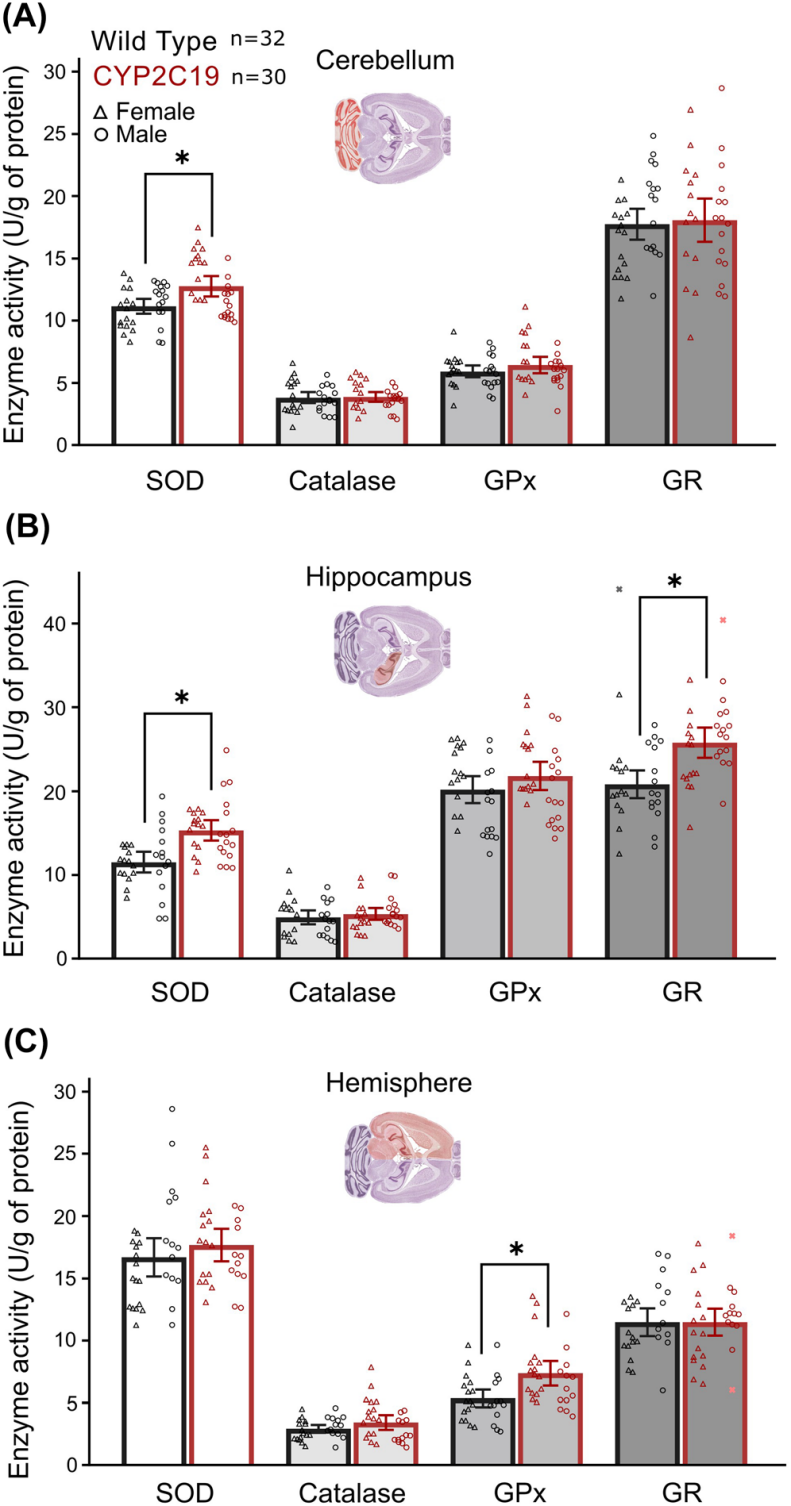
Antioxidant enzyme activity in three brain regions. Compared with wild‐types, *CYP2C19* transgenic mice exhibited (A) increased superoxide dismutase (SOD) activity in the cerebellum (1.14‐fold [95% CI: 1.06, 1.23], *F*_1,60_ = 7.0, *p* = 0.0010, *q* = 0.023), (B) increased hippocampal activity of SOD (1.3‐fold, [95% CI: 1.18, 1.47], *F*_1,59_ = 18.7, *p* < 0.0001, *q* = 0.0013) and glutathione reductase (GR, 1.2‐fold [95% CI: 1.13, 1.35], *F*_1,58_ = 17.5, *p* < 0.0001, *q* = 0.0021) and (C) increased activity of glutathione peroxidase (GPx) in the whole hemisphere (1.38‐fold [95% CI: 1.16, 1.59], *F*_1,57_ = 11.9, *p* = 0.0011, *q* = 0.023). Graphic representations of the brain were adopted from the reference atlas [27]. Data presented as means with whiskers as 95% CI or as Tukey box‐plot. Bars and whiskers represent means and 95% CI. *Statistical significance p < 0.05.

Next, to indirectly investigate the presence of excessive oxidative stress in the cerebellum and hippocampus, regions altered in *CYP2C19* transgenic mice, we measured the expression and activity of antioxidative enzymes in them, namely, SOD, CAT, GPx and GR (Figure 3). Several samples have been excluded from the analyses due to invalid measurements (Supporting Information), mostly due to insufficient sample volumes. The activity of SOD was slightly increased in the cerebellum (Figure 3A) and moderately increased in the hippocampus (Figure 3B) of transgenic mice as compared with the *wild‐types*. In addition, there was a significant increase in GR enzyme activity in the hippocampus of transgenic mice (Figure 3B). Finally, there were no significant changes in the expression levels of any enzyme in the investigated brain regions (Supporting Information). Detailed analysis outputs are presented in the Supporting Information. In summary, the *CYP2C19* transgenic mice exhibit normal expression but higher antioxidative enzymatic activity in the cerebellum and hippocampus compared with *wild‐types*.

**FIGURE 4:**
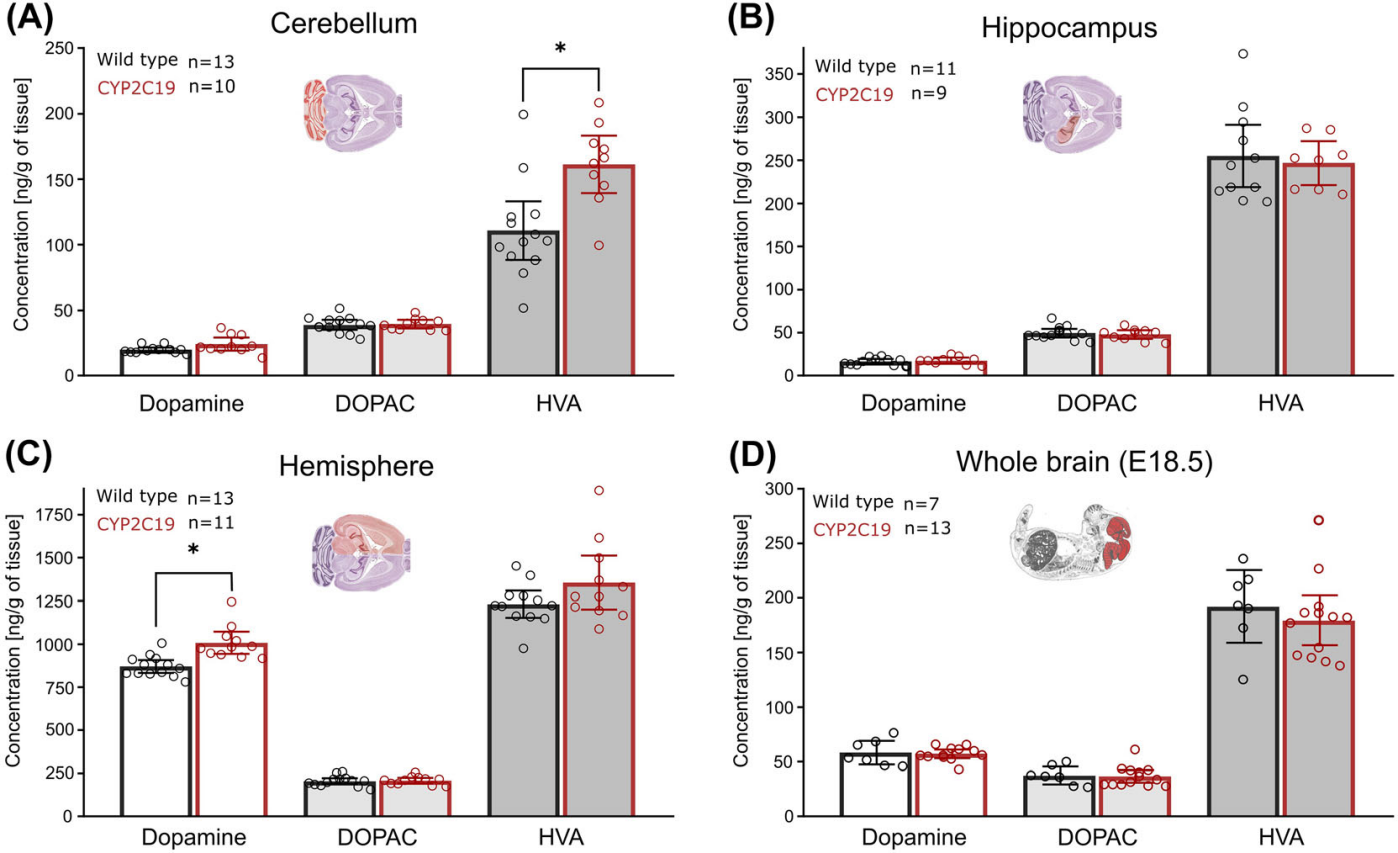
*CYP2C19* mice exhibit hyperdopaminergism in adulthood but not during development. There was an initial statistical trend towards 21% (*t*_20_ = −1.95, *p* = 0.065) increased dopamine concentrations in the cerebella of *CYP2C19* mice, which was no longer present after p‐value correction (*q* = 0.45). Also, homovanillic acid (HVA) level was 46% ([95% CI: 21%, 71%], *t*_21_ = −3.52, *p* = 0.0020, *q* = 0.016) increased in *CYP2C19* mice compared with controls (A). Hippocampal levels of dopamine, 3,4‐dihydroxyphenylacetic acid (DOPAC) and HVA were not different between test groups (B). *CYP2C19* transgenic mice were hyperdopaminergic with slightly increased (1.15‐fold [95% CI: 1.08, 1.23]; *t*_22_ = −4.26, *p* = 0.0003, *q* = 0.0029) dopamine concentrations in the hemisphere compared to *wild‐types* (C). Graphic representations of the brain [27] and embryo [28] were adopted from reference atlases. Bars and whiskers represent means and 95% CI. *Statistical significance p < 0.05.

**FIGURE 5:**
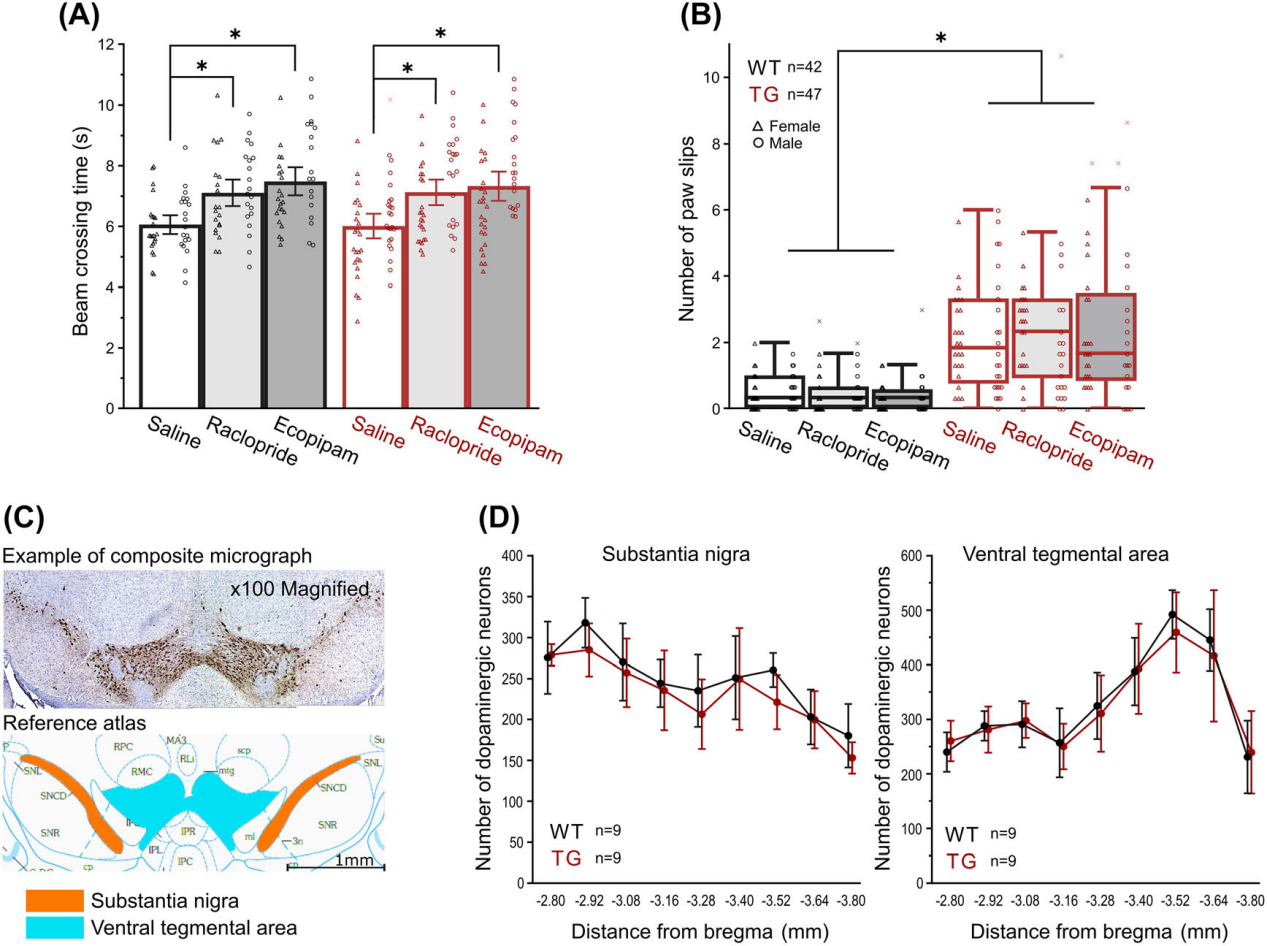
The dopaminergic system is not associated with motoric impairment in *CYP2C19* transgenic mice. Dopaminergic antagonists failed to ameliorate motoric impairment in *CYP2C19* transgenic mice in the beam‐walking test, and they in fact slightly prolonged beam walking time (Ecopipam: 1.2‐fold [95% CI: 1.17, 1.30], *F*_1,76_ = 13.9, *p* < 0.0001; raclopride: 1.2‐fold [95% CI: 1.12, 1.25], *F*_1,76_ = 13.9, *p* < 0.0001) in them (A, B). Anti‐tyrosine hydroxylase (TH) immunohistochemistry (C) revealed no significant changes in the numbers of midbrain TH+ neurons (D) after false discovery rate (FDR) correction. Data presented as means with whiskers as 95% CI or as Tukey box‐plot. *Statistical significance p < 0.05.

Finally, the structure and function of the dopaminergic system in *CYP2C19* transgenic mice were investigated. While there were no changes in levels of either dopamine or its metabolites in *CYP2C19* mice embryos at day E18.5 (Figure 4D), adult *CYP2C19* transgenic mice exhibited a 1.15‐fold increase in the brain hemisphere dopamine concentration compared with *wild‐type*s (Figure 4C). The concentration of HVA in the cerebellum was 1.46‐fold higher in *CYP2C19* mice compared with controls, whereas cerebellar dopamine concentrations showed only a nominal statistical trend towards higher levels in *CYP2C19* animals; this trend was no longer present after correction for multiple testing (Figure 4A). To investigate the effect of D1 antagonist ecopipam and D2 antagonist raclopride on the previously observed motor phenotype of hyperdopaminergic *CYP2C19* transgenic mice, animals repeated BW tests shortly after the acute drug treatments. Both drugs failed to ameliorate the motor function impairment of *CYP2C19* transgenic mice; in contrast, they slightly prolonged beam crossing time in all agonist‐treated compared with saline‐treated mice, without genotype‐specific changes (Figure 5A). To investigate the potential changes in the shape and microstructure of dopaminergic nuclei, the number of tyrosine hydroxylase positive (TH+) neurons was quantified throughout the rostro‐caudal axis. Because the mouse age did not affect the number of dopaminergic neurons and because it did not interact with genotype, this covariate was excluded from the main analysis, and 6 and 15 months old mice were considered as a homogenous group and analysed together. In the section, −3.52 mm from bregma, the number of dopaminergic neurons in SN was significantly (1.15‐fold) reduced in the *CYP2C19* transgenic mice, compared with *wild‐types* (Figure 5D); however, this change did not remain significant after multiple comparisons correction. Finally, neuromelanin staining was negative in dopaminergic neurons in the SN in all mice, unrelated to the age and genotype. In summary, *CYP2C19* transgenic mice exhibit mild hyperdopaminergism and increased cerebellar dopamine turnover, which is unlikely the cause behind the observed motor phenotype and which is not accompanied by any pronounced structural changes within dopaminergic nuclei.

## DISCUSSION

*CYP2C19* transgenic mice exhibit altered gait and slightly worse performance in more demanding motoric tasks, including impaired balance. This motoric phenotype shares several attributes of ataxia arguing for the face validity of the model; because abnormal walking, diminished balance and loss of fine dexterity are indeed some of the most commonly described symptoms of cerebellar ataxia [1]. Of note, the absence of a wider hind base on the footprint test, non‐progressive nature, unilateral manifestation and mild intensity does not resemble commonly observed symptoms of cerebellar ataxia in humans, but our model may still be of relevance for certain disease subtypes.

Another important ataxia‐like characteristic of *CYP2C19* mice is the presence of cerebellar atrophy in adulthood, which is in concordance with neuroimaging observations in humans with ataxia [1]. Therefore, it is quite likely that cerebellar atrophy is one of the causes of the observed motoric phenotype in *CYP2C19* transgenic mice. Atrophy was also observed in the hippocampus confirming the previous results [6]; the only difference being that Persson et al. reported a reduction of 7.1% in contrast to the reduction of 4.2% observed here. This discrepancy most probably resulted from differences in tested cohorts; Persson et al. included seven male animals per group, whereas this study included 15 male and 15 female animals per group. Volumetric alterations observed in both regions likely originate from aberrant development but may also occur due to the proneness to atrophy of the affected structures after their development is completed. Increased activities of antioxidant enzymes in the cerebellum and hippocampus of adult *CYP2C19* transgenic mice in this study indeed hint at increased oxidative stress exposure and potential proneness to excitotoxicity and neuronal apoptosis in these regions.

Another possible cause for motoric impairment in *CYP2C19* mice investigated was the hyperdopaminergism, but this is unlikely as antidopaminergic drugs failed to ameliorate the motoric impairments of transgenic mice. Cerebellar dysfunction can lead to the compensatory hyperactivation of dopamine transmission in basal ganglia [29, 30], which is most likely the case with the *CYP2C19* mouse model as well. Profoundly increased levels of HVA in the cerebella of *CYP2C19* mice are likely associated with altered emotionality rather than locomotion in this model as a recent in vivo rodent study revealed association with dopaminergic innervation in a cerebellum and social behaviour alterations but not motoric alterations [31]. Also, hyperdopaminergism was not associated with dopaminergic cell loss in the midbrain in both adult and older *CYP2C19* transgenic mice, despite hypothetically increased vulnerability of dopaminergic neurons to cell death due to chronic hyper‐activation [32]. Even though neuromelanin aggregates are not normally detectable in rodents due to their relatively short lifespans compared with the long process of neuromelanin production [33], a few animal models with altered dopaminergic *nigro‐striatal* pathways have been described to exhibit detectable neuromelanin levels [33, 34]. However, neuromelanin was not detected in 15 months old *CYP2C19* transgenic mice in this study, arguing that the increase in dopamine production was not sufficient to cause quantifiable neuromelanin aggregation in this particular transgenic mouse.

Several animal models exhibit a motoric phenotype similar to the one observed in *CYP2C19* transgenic mice [35, 36, 37]. For example, Atcay^ji‐hes^ mouse exhibits more extreme gait disturbances and involuntary hind‐paw movements while walking, which were not observed in the *CYP2C19* transgenic mice (Movie 2 from Luna‐Cancalon et al. [35]). Next, the *Reeler* mouse can be considered as the closest phenocopy of the *CYP2C19* transgenic mouse, as it has a very similar gait (Video S1 in Machado et al. [36]); this behaviour is also accompanied by impaired performance in rotarod and BW tests. *Reeler* mice also exhibit an aberrant formation of the hippocampus and profoundly reduced cerebellar size [36]. Finally, Sgce^m+/pGt^ mice, besides similar gait to *CYP2C19* transgenic mice, also exhibit slight tremors, more slips in the BW test and abnormal expression of several genes in the cerebellum that is potentially associated with aberrant cerebellar development [37]. Because none of the mentioned models gives detailed enough reports of the circuitry involved in their phenotype, it is hard to extrapolate which motoric pathway is affected in *CYP2C19* transgenic mice. Still, all phenotypically similar models exhibit abnormal cerebellar development and/or reduced output from Purkinje cells suggesting that the same might be true in the *CYP2C19* transgenic mouse model, but further research is needed to investigate this. Additionally, *CYP2C19* transgenic mice exhibit a clasping reflex that, besides being a non‐specific symptom, can be narrowed down to disturbances in either cerebello‐cortico‐reticular or cortico‐striato‐pallido‐reticular pathway [12] and that can be triggered by alterations in either noradrenergic or serotonergic transmission. Because the *CYP2C19* model has cerebellar atrophy and abnormal serotonergic system activity [5], it can be speculated that the clasping reflex in *CYP2C19* transgenic mice is caused by cerebello‐cortico‐reticular pathway disturbance combined with abnormal serotonergic system function, but further research on this topic is needed for firmer claims.

Importantly, *CYP2C19* transgenic mice possess a few unique properties, not previously observed in any other animal model of ataxia: (1) *CYP2C19* transgenic mice either stagnate or spontaneously improve phenotypic characteristics, whereas in most other ataxia models, progressive worsening of motoric function is observed [4]; (2) Although most animal models of ataxia exhibit altered movements in both sides of the body [4], only one side of the body is affected with motoric impairment in the *CYP2C19* transgenic mice in their later adulthood; (3) Although many animal models of ataxia exhibit wider hind base on a footprint test, *CYP2C19* transgenic mice nearly do not exhibit abnormalities in footprint test parameters. In conclusion, *CYP2C19* transgenic mice show a motoric phenotype that shares some, but not all, ataxia‐like features and could be useful in the in vivo investigations of certain aspects of cerebellar function and development. Understanding the exact molecular mechanisms involved in *CYP2C19* transgenic mouse motoric phenotype, including the processes behind the spontaneous improvement in some aspects of the motoric phenotype in young *CYP2C19* transgenic mice, could provide insight into new drug targets for the treatment of cerebellar disorders.

### Limitations

Most importantly, the time course for several observed effects is still virtually unknown; measurements of brain morphology at different time points would be necessary to determine if the changes occurred during development or after birth and if the latter is the case, when exactly. Next, *CYP2C19* mice are more susceptible to stress, and it cannot be excluded that the usage of a light source in the BW and footprint test to motivate animals to complete the tasks could affect two test groups asymmetrically; however, the animals were exposed to several training sessions, and they were habituated to the light, and it is, therefore, unlikely that the light as a stressor affected the result in a meaningful way. Next, antioxidative enzyme activity and expression only provide information related to the general state in the observed regions; detection of ROS or oxidative damage is still needed to confirm the presence of disrupted oxidative/antioxidative balance in brain regions of interest.

## CONFLICTS OF INTEREST

All authors declare no conflict of interest.

## ETHICS STATEMENT

All laboratory animal experiments were performed according to the permit of the Ethical Committee on Animal Experimentation of the University of Belgrade – Faculty of Pharmacy, Serbia (permit number 23‐07‐00933/2019‐05 to MMJ).

## AUTHOR CONTRIBUTIONS

FM conducted motoric tests and sample collecting, coded and statistically analysed all data except VBM and wrote the original manuscript draft. IB designed and conducted VBM experiment and analysed VBM data. AA designed and conducted footprint and gait analysis. MM and MV contributed in design and execution of behaviour tests and sample collection. ZOD, JB, ANK and DB designed and conducted oxidative status experiments. ČM contributed in study design and conceptualisation. CW and PD designed and conducted neuroimaging experiment; VP and MIS contributed in study design and conceptualisation and funding acquisition. RFT contributed in study design and conceptualisation and designed dopamine concentration measurement experiment. MMJ managed study execution and contributed in study design and conceptualisation and funding acquisition. All authors critically reviewed and approved the manuscript.

### PEER REVIEW

The peer review history for this article is available at https://publons.com/publon/10.1111/nan.12867.

## Supporting information


**Figure S1:** Demonstration of the parameter measurements in footprint test
**Table S1:** Protocol of pharmacological treatment in the beam walking test
**Table S2:** Significance and magnitude of the impact of the genotype on the study results
**Table S3:** Significance of the impact of sex as a covariate in the study results
**Table S4:** Significance of the impact of age as a covariate in the study results
**Table S5:** Frequencies of TG animals with unilateral or bilateral motoric phenotypes
**Figure S2:** Footprint analysis: Stride lengths for every paw in young and adult mice.
**Figure S3:** Expression of antioxidant enzymes in 3 brain regions.

**Data S1.** Supporting Information

**Video S1:** **Ataxia‐like gait in *CYP2C19* transgenic mouse (.mp4)**

A *wild‐type* mouse was used as a control for the comparison of the gait disturbance in an adult (>10 weeks old) *CYP2C19* transgenic mouse, and a more pronounced disturbance in 3 weeks old transgenic mouse.

**Video S2:** ***CYP2C19* transgenic mouse exhibits significantly more slips in the beam walking test (.mp4)**

Representative baseline beam‐walking runs of one wild type and one *CYP2C19* transgenic mouse are shown. The criterion for the counting of the paw slips is also demonstrated.

## Data Availability

The data that support the findings of this study are available in the Supporting Information of this article.
