# Supplementary material for: The humanised CYP2C19 transgenic mouse exhibits cerebellar atrophy and movement impairment reminiscent of ataxia
Source: Neuropathol Appl Neurobiol. 2023 Jan 17;49(1):e12867. doi: 10.1111/nan.12867 (PMC10108232; doi:10.1111/nan.12867)
Supplement: Supplementary file 1 — Figure S1: Demonstration of the parameter measurements in footprint test Table S1: Protocol of pharmacological treatment in the beam walking test Table S2: Significance and magnitude of the impact of the genotype on the study results Table S3: Significance of the impact of sex as a covariate in the study results Table S4: Significance of the impact of age as a covariate in the study results Table S5: Frequencies of TG animals with unilateral or bilateral motoric phenotypes Figure S2: Footprint analysis: Stride lengths for every paw in young and adult mice. Figure S3: Expression of antioxidant enzymes in 3 brain regions. [file NAN-49-0-s002.docx]

Supplementary material to:

**The humanized *CYP2C19* transgenic mouse exhibits cerebellar atrophy and movement impairment reminiscent of ataxia**

Filip Milosavljević^1^, Irene Brusini^2,3^, Andrea Atanasov^1^, Marina Manojlović^1^, Marija Vučić^1^, Zorana Oreščanin Dušić^7^, Jelena Brkljačić^7^, Čedo Miljević^8,9^, Aleksandra Nikolić^7^, Duško Blagojević^7^, Chunliang Wang^2^, Peter Damberg^10^, Vesna Pešić^1^, Rachel F Tyndale^4,5,6^, Magnus Ingelman-Sundberg^11^, and Marin M Jukić^1,11^

1. Department of Physiology, Faculty of Pharmacy, University of Belgrade, Serbia
2. Department of Biomedical Engineering and Health Systems, KTH Royal Institute of Technology, Huddinge, Sweden;
3. Department of Neurobiology, Care Sciences and Society, Karolinska Institute, Solna, Sweden.
4. Campbell Family Mental Health Research Institute, Centre for Addiction and Mental Health, Toronto, Ontario, Canada.
5. Department of Psychiatry, University of Toronto, Ontario, Canada.
6. Department of Pharmacology and Toxicology, University of Toronto, Ontario, Canada.
7. Institute for Biological Research "Siniša Stanković", Belgrade, Serbia.
8. Department of Psychiatry, Faculty of Medicine, University of Belgrade, Serbia
9. Institute for Mental Health, Belgrade, Serbia
10. Karolinska Experimental Research and Imaging Center, Karolinska University Hospital, Solna, Sweden.
11. Section of Pharmacogenetics, Department of Physiology and Pharmacology, Karolinska Institutet, Stockholm, Sweden

**Corresponding author:**

Marin M Jukić, MPharm PhD, University of Belgrade – Faculty of Pharmacy (Vojvode Stepe 450, 11221 Belgrade, Serbia; [marin.jukic@pharmacy.bg.ac.rs](mailto:marin.jukic@pharmacy.bg.ac.rs)) and Karolinska Institutet (SE-171 77 Stockholm, Sweden; [marin.jukic@ki.se](mailto:marin.jukic@ki.se))

Table of Contents

[1. METHODS 3](#_Toc118298924)

[**1.1. Laboratory animals** 3](#_Toc118298925)

[**1.2. Footprint test** 3](#_Toc118298926)

[**Figure S1:** Demonstration of the parameter measurements in footprint test 4](#_Toc118298927)

[**1.3. Paw elevation height** 4](#_Toc118298928)

[**1.4. Clasping reflex screening** 5](#_Toc118298929)

[**1.5. Rotarod test** 5](#_Toc118298930)

[**1.6. Beam-walking test** 6](#_Toc118298931)

[**1.7. Measurements of Dopamine Concentration** 6](#_Toc118298932)

[**1.8. Anti-dopaminergic Drugs and Motoric Performance** 7](#_Toc118298933)

[**Table S1:** Protocol of pharmacological treatment in the beam walking test 8](#_Toc118298934)

[**1.9. Immunohistochemistry** 8](#_Toc118298935)

[**1.10. Neuromelanin detection:** 9](#_Toc118298936)

[**1.11. Gadolinium-enhanced Neuroimaging** 9](#_Toc118298937)

[**1.12. Antioxidant Enzyme Activity** 11](#_Toc118298938)

[**1.13. Antioxidant Enzyme Expression** 11](#_Toc118298939)

[**1.14. Statistics** 12](#_Toc118298940)

[2. RESULTS 14](#_Toc118298941)

[**2.1. Tabular representation of genotype specific results of the study** 14](#_Toc118298942)

[**Table S2:** Significance and magnitude of the impact of the genotype on the study results 14](#_Toc118298943)

[**2.2. Effects of covariates** 20](#_Toc118298944)

[**Table S3:** Significance of the impact of sex as a covariate in the study results 20](#_Toc118298945)

[**Table S4:** Significance of the impact of age as a covariate in the study results 24](#_Toc118298946)

[**2.3. Determination of the cohort size** 25](#_Toc118298947)

[**2.4. Bilaterality of abnormal hindpaw elevation in *CYP2C19* transgenic mice** 27](#_Toc118298948)

[**Table S5:** Frequencies of TG animals with unilateral or bilateral motoric phenotypes 27](#_Toc118298949)

[**2.5. Stride lengths in young and adult *CYP2C19* transgenic (TG) and *Wild type* (WT) mice** 28](#_Toc118298950)

[**Figure S2:** Footprint analysis: Stride lengths for every paw in young and adult mice. 28](#_Toc118298951)

[**2.6. Expression of the anti-oxidative enzymes in the brain tissue of *CYP2C19* transgenic mice** 29](#_Toc118298952)

[**Figure S3:** Expression of antioxidant enzymes in 3 brain regions. 30](#_Toc118298953)

[3. REFERENCES 31](#_Toc118298954)

# 1. METHODS

## **1.1. Laboratory animals**

General signs of animal wellbeing that were monitored included level of activity, fur condition and weight loss. Signs of abnormal behavior that were monitored were repetitive movements, inability to complete the training and lack of motivation to complete experimental tasks. Animal wellbeing was checked minimum twice a week, but during the pharmacological treatments, wellbeing and the signs of distress were checked every day. Behavioral tests were performed during the light-phase and animals were being accustomed to the testing room for at least 30 minutes. Diluted (10% v/v) ethanol was used to clean apparatus between the runs to eliminate the smell of a previous tested animal that could interfere with the test, while avoiding behavioral effects of inhaled ethanol.

Body weight was measured at the same time of the day: between 11am and 2pm.

## **1.2. Footprint test**

In young animals, footprint analysis was performed once a week from 5^th^ until 8^th^ postnatal week with the total of 4 time points. Adult mice 7-9 months old were also tested at one time point. Apparatus used in the footprint test comprised of 6 cm wide and 100 cm long runway with and 15 cm high transparent clirit walls. Strong light source was placed at the starting position on the runway while the dark closed box was placed at the end; this way, mice were motivated to cross the runway and escape from the stress-inducing stimulus and enter the closed area which mice instinctively consider safe as a pray animal. Mice were trained one week before the main experiment. In a training run, mice were first left to explore the runway freely, and the training was considered successful once and animal stated running across the runway at a steady pace and in a straight line. For the main experiment, new sheet of white paper was placed on the floor of the runaway for each mouse and fore and hind paws of the mice were coated with red and green, non-toxic, washable paint, respectively. The mouse was then held at the entrance of the runaway and gently released. Run was successful if footprints from at least 4 uniform steps could be collected, excluding footprints of proximal and distal ends since they frequently show large variations. Distance between two footprints was measured with a ruler from the center of one footprint paint blot to the center of the other blot. The step parameters measured were stride length (the distance between consecutive footprints made by the same paw) for each paw, front and hind base width (the distance between left and right footprints of the two forepaws or two hindpaws) and overlap between forelimb and hindlimb (the distance between pads of left (or right) front and hind footprints):


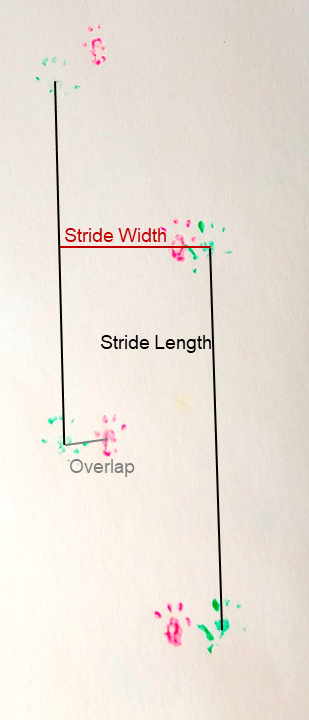


### **Figure S1:** Demonstration of the parameter measurements in footprint test

Average of three values for each parameter collected from the 4 representative steps was calculated and compared between the groups.

## **1.3. Paw elevation height**

Height of the hind paw elevation was measured on the stills of sideways video footage of the footprint test runs on the same animal cohort. Digital camera (Canon PSA3100IS, Japan; Video quality: 640x480px, 30fps) was placed 18.5cm from the apparatus at the same position and at the same height from the ground in every run. Since animals ran across the narrow runway and since the only steps that happened at the center of the frame were considered for the analysis, it was assumed that all animas were at the same distance from the camera. Video footage was searched frame by frame in order to detect the point at which the hind paw closer to the camera was in the uppermost position of the step cycle. Stills of the video material collected this way were analyzed with ImageJ freeware software (ImageJ 1.53, NIH, USA) where the number of pixels between the lowest edge of the paw and the surface was measured and compared across groups.

## **1.4. Clasping reflex screening**

Flexion of animal’s limbs or body or clinching of an animal’s paws together were used as visual signs of the clasping reflex, and both were induced once an animal was suspended by its tail and then slowly lowered towards the hard surface. On the other hand, extension of limbs in the anticipation of the fall was considered a physiological reaction^1^.

## **1.5. Rotarod test**

In the *rotarod* test, Ugo Basile Rotarod 47700 (Ugo Basile, Italy) apparatus with four 8.7 cm wide lanes was used. Animals were trained to run against the rotating rod where the rotation speed was gradually increased until it became impossible for any mouse to keep the balance and stay on the rod. Two training trials were done on the two consecutive days with 3 runs per trial, and the animals that were unable to learn the task were excluded from the experiment. Every training run consisted of a 2-minute walk on a rod rotating at low speed of 5 RPM (revolutions per minute) with 5 minute breaks in the home cage between the runs. On the third day, final trial was performed and the data was collected. Animals were placed on the rod rotating at 2 RPM and were allowed to take a proper position, after which the test run was started and the rod began to accelerate at the rate of +8RPM per minute (2-50 RPM in 6 minutes). Falls were detected automatically by trip-switches under the rod; but if an animal grabbed onto the rod to avoid falling, the run was manually stopped at the moment when the animal stopped running. Six runs were performed with at least 5 minute break in a home cage between the runs. Run was considered failed if an animal fell from the apparatus for any other reason besides failing to keep up with the rotation speed. If an animal had 3 or more failed runs, it was excluded from the experiment due to the lack of motivation.

## **1.6. Beam-walking test**

In the beam-walking test, rectangular, 8mm wide and 1m long transparent acrylic beam was used. Strong light source was placed at the starting position on the beam in order to induce the anxious response in tested mice, and on the opposite end there was closed dark box which served as a goal, since mice have a natural tendency for seeking shelter in closed and dark spaces. Beam was also set at the slight incline since mice tend to seek high ground when escaping from a danger^2^. Mice were also allowed to accommodate to the dark box for 1 minute before every run in order to facilitate the feeling of safety inside the dark box. Animals were also trained to cross the beam for 2 consecutive days before the experiment. On the day 1, mice were placed 25cm, 50cm and 75cm away from the goal in the first, second and third training run, respectively; and in every run, mice were allowed to explore and find the way to the goal box. On the day 2, mice were allowed to cross the full length of the beam, and if an animal could not finish the task 3 times it was excluded from the experiment. On the third day, 5 test runs were performed with the additional sixth run, if at least one run failed. Run was considered failed if an animal slowed down or completely stopped while crossing the beam; if there were less than 3 valid runs in total, animal was excluded from the experiment due to the lack of motivation to perform in this task. Every run was filmed for later analysis and beam crossing time was measured live using stopwatch, and validated later upon the analysis of the videos. Two researchers independently determined number of slips by reviewing the video footage, and one slip was defined as the failure to keep the paw on the beam while trying to make one step.

## **1.7. Measurements of Dopamine Concentration**

Concentration of dopamine was determined in the brain hemispheres of 3 months old mice using HPLC-MS-MS method. Extraction solution (15% methanol and 15% acetonitrile in water adjusted to pH=2.5 using acetic acid) was added to the harvested mouse brains in the volume of 0.009 ml/mg of tissue followed by homogenization. Dopamine-*d4*, HVA-*d2*, DOPAC-*d5* were added to the homogenate as an internal standard in the amount of 1 ng/mg each of tissue followed by centrifugation at 11,000g. Twenty-five µl of supernatant was injected into the Agilent 1260 LC system containing (1) Agilent 1260 Quaternary pump set to flow rate of 0.200 ml/minute, (2) Agilent 1260 Infinity Standard Autosampler and (3) temperature-controlled column compartment that was connected to (4) Agilent 6430 QQQ. Gemini 5 µ C18 110 Å 150 x 2 mm column (Phenomenex, USA) was used and the mobile phase consisted of Solvent A (aqueous solution of acetic acid, pH 2.5) and Solvent B (methanol). The following gradient elution was used: 5% B 0 - 3 minutes, 3 - 5 minutes linear increase to 70% B, maintained 5-15 minutes, 15 - 16 minutes linear decrease to 5% B, maintained 16- 25 minutes. The QQQ was equipped with an electrospray ion source and operated in positive ion mode for the purpose of measuring dopamine with monitored transitions being (eV) 154 ⇒ 137 for Dopamine, 158 ⇒ 141 for Dopamine-d4, 181 ⇒ 122 for HVA; 183 ⇒ 124 for HVA-d2, 167 ⇒ 122 for DOPAC; 172 ⇒ 127 for DOPAC-d5. The calibration standards prepared in the extraction solution had concentration range of 5 - 1000 ng/ml ^3^.

Noteworthy, this was the only experiment which included only males, since the females had to be spared for the breading and colony expanding purpose at the time this experiment was conducted.

## **1.8. Anti-dopaminergic Drugs and Motoric Performance**

Beam walking test was repeated after the treatment with selective D1 antagonist ecopipam (SCH-39166, Tocris Bioscience, UK) and D2 antagonist raclopride (Tocris Bioscience, UK). Animals in the both test groups were divided into three subgroups in a manner to ensure that the beam crossing time is equivalent between the subgroups. Each subgroup received saline, raclopride, or ecopipam prior to the retesting. There were three beam walking test trials with 7-day washout period between them, so that each animal could receive every respective treatment, as shown in the Table S1:

### **Table S1:** Protocol of pharmacological treatment in the beam walking test

| Test groups (females/males) | Trial 1 |  | Trial 2 |  | Trial 3 |
| --- | --- | --- | --- | --- | --- |
| Group 1 n=30  *CYP2C19* transgenic n=17 (9/8)  *Wild-Type* n=13 (7/6) | Saline | 7-day washout | Raclopride | 7-day washout | Ecopipam |
| Group 2 n=30  *CYP2C19* transgenic n=16 (8/8)  *Wild-Type* n=14 (8/6) | Raclopride |  | Ecopipam |  | Saline |
| Group 3: n=29  *CYP2C19* transgenic n=15 (8/7)  *Wild-Type* n=14 (7/7) | Ecopipam |  | Saline |  | Raclopride |

The same beam walking test protocol was used as previously described, but without the training phase, since the animals already had previous experience with the beam walking test.

## **1.9. Immunohistochemistry**

Adult mouse brain samples from transgenic and control mice were fixed using 4% formaldehyde and embedded into paraffin blocks. Tissue samples were then cut into 10µm thick coronal sections using Leica RM 2125RT microtome (Leica Biosystems, Germany) that were collected on the Superfrost® Plus microscope slides (Thermo-Fisher Scientific, Germany). Initially, slides were deparaffinized by xylene and then rehydrated throughout the series of ethanol solutions of decreasing concentrations, followed by a brief rinse in the distilled water. From this point on, slides were rinsed with the PBS (Phosphate-buffered saline, 0.1M, pH=7.4) solution 3 times for 5 minutes between steps. Antigen retrieval was done by heating slides submerged into citrate buffer to its boiling point in the microwave oven for 20 minutes. To reduce unspecific staining due to endogenous peroxidase activity, slides were incubated with the 2% H_2_O_2_ solution for 10 minutes. Next, the slides were covered with blocking solution (2.5% Normal Horse Plasma, Vector Laboratories, USA) for 30 minutes to block potential signaling from nonspecific tissue epitopes. Next, slides were incubated overnight at +4°C with rabbit anti-Tyrosine Hydroxylase antibody (1:250 in PBS, ab137869, Abcam, UK). On the next day, slides were incubated with secondary horseradish peroxidase conjugated antibody (ImmPRESS™ Anti Rabbit Ig Reagent, Vector Laboratories, USA) for 2h. Specific signal was developed by using peroxidase substrate Diaminobenzidine (DAB substrate kit, ab64238, Abcam, UK) followed by brief counterstaining with haematoxylin stain. Representative slides were -2.80mm, -2.92mm, -3.08mm, -3.16mm, -3.28mm, -3.40mm, -3.52mm, -3.64mm and - 3.80mm from bregma (Figures 54, 55, 56, 57, 58, 59, 60, 61 and 62, respectively, from mouse brain atlas^4^. Stained slides were observed under the BX50 light microscope (Olympus System, Japan) with 3CCD Color Video Camera DXC-950P (Sony, Japan) mounted on the top. Composite micrographs were compared to the referent atlas in order to determine the boundaries of SN and VTA on the given section^4^.

## **1.10. Neuromelanin detection:**

Presence of neuromelanin was assessed on 10µm thick sections of paraffin embedded mid-brains of 15-month-old transgenic and control mice stained with Fontana-Masson melanin stain (ab150669, Abcam, UK). In short, paraffinized sections were rehydrated and incubated at 60°C for about 40 minutes in freshly prepared ammoniac silver nitrate solution. Next, slides were incubated first with 0.2% gold-chloride solution for 30 seconds, then with sodium-thiosulfate for 2 minutes and finally with nuclear Fast Red stain for 5 minutes at room temperature with rinsing in distilled water before every step. Stained slides were then dehydrated, mounted and observed under the BX50 light microscope (Olympus System, Japan). Presence of black granules in neurons was considered as a sign of neuromelanin aggregation.

## **1.11. Gadolinium-enhanced Neuroimaging**

Gadolinium (Gd) containing contrast agent ProHance (0.5M Gadoteridol, Bracco diagnostics, USA) was used to increase the contrast during the MRI scans and consequently increase the resolution of the obtained 3D images. To prepare the brain tissue for neuroimaging animals were first overdosed with anesthetic, fixed on the operation table, and thoracic cavity was opened in order to expose animal’s heart. Needle connected to the tubing of the peristaltic pump (LabKemi, Sweden) was inserted into left ventricle, and the cut was made on the right ventricle for the fluid outflow. Using the pump, whole animal was transcardially perfused first with 5 ml of PBS solution in order to replace the blood and prevent clothing, followed with 10 ml of Gd-enriched fixating solution (11.1% v/v of 36% formaldehyde solution and 10% v/v of contrast agent in 0.1 M phosphate buffer solution, pH=7.4) which fixed the brain tissue and saturated brain extracellular fluid with Gd contrast agent. At the end of the perfusion, animals were decapitated and soft tissue, and extra-cranial tissue was removed. Harvested samples were kept in Gd enriched fixating solution overnight, followed by the transfer into gadolinium enriched PBS (1% v/v of ProHence in 100 ml of 0.1 M PBS, pH=7.4) in which samples were kept at +4°C until the MRI scan. Shortly before the structural 9.4 T MRI scan, four heads were wrapped in gauze and placed in a 30 ml syringe. The void of the syringe was filled with perfluorinated oil, i.e. fomblin (Solaway Solexis, NJ, USA), as a non-signaling susceptibility matched environment for the heads.

Medulla oblongata was also excluded due to possible damage to the tissue caused by decapitation of an animal. Volumes were analyzed for the following 20 regions of interest: Amygdala, Cerebellum, Cerebral Cortex, Corpus Striatum, Epithalamus, Globus Pallidus, Hippocampal Formation, Hypothalamus, Inferior Colliculus, Interpeduncular Nucleus, Nucleus Accumbens, Olfactory Bulb, Periaqueductal Gray, Pineal Gland, Pons, Septal Nucleus, Substantia Nigra, Superior Colliculus, Thalamus, Unsegmented Midbrain.

As of voxel-based morphometry, we had to generate a new study-specific mouse GM template, which was obtained by non-linearly registering all GM segmentations to the segmentation of one mouse, randomly chosen from the dataset, and averaging them. Afterwards, the original VBM pipeline was closely followed by registering all native GM images to the study-specific template and “modulating” them to correct for local expansion or contraction. The modulated GM images were then smoothed with an isotropic Gaussian kernel with a sigma of 0.3 mm.

## **1.12. Antioxidant Enzyme Activity**

Tris buffer (50 mM Tris, 0.25 M sucrose, 1 mM EDTA, pH 7.4) has been added to the flash frozen tissue in the volume of 0.1 ml/g of tissue prior to the homogenization by sonification (3 x 10s at 10 MHz on ice). This mixture was centrifuged at 105,000 g for 60 min and collected supernatant was stored in liquid nitrogen until use. The protein concentration was determined by the Lowry method^5^, and bovine serum albumin was used as standard. Total SOD activity was determined by the adrenaline method^6^ and SOD unit was defined as the catalytic activity that decreases the rate of adrenalin auto-oxidation by 50% at pH=10.2. CAT activity was determined according to Beutler^7^ and CAT unit was defined as the activity sufficient to decompose 1 mmol H_2_O_2_ per minute at 25°C and pH 7.0. The activity of GPx was determined by the glutathione reduction of t-butyl hydroperoxide, using the modification of the assay described by Paglia and Valenine^8^ and GR activity was determined using the method of Glatzle^9^. GPx unit was defined as the activity that oxidizes 1 mmol of NADPH per minute at 25°C and pH 7.0, and GR unit was defined as an enzyme activity that oxidizes 1nmol of NADPH per minute at 25°C and pH 7.4.

## **1.13. Antioxidant Enzyme Expression**

Western blot method for specific protein quantification was done by boiling of tissue samples in Laemmli's sample buffer, followed by protein resolution on the 10% or 12% SDS polyacrylamide gels. Western transfer of proteins from the gels to PVDF membranes was performed at 135 mA overnight in the 25 mM Tris buffer, pH=8.3 containing 192 mM glycine and 20% v/v methanol. The membranes were blocked with the 5% non-fat dry milk in PBS (1.5 mM KH_2_PO4, 6.5 mM Na_2_HPO4, pH=7.2, 2.7 mM KCl, 0.14 M NaCl) for 1h at room temperature. After blocking, membranes were incubated overnight at 4 °C with the respective primary antibody: SOD1 (ab13498, 1:5000), SOD2 (ab13533, 1:2000), CAT (ab16731, 1:2000), GPx (ab22604, 1:2000), GR (ab16801, 1:2000), and β actin (ab8227, 1:5000). Membranes were subsequently washed with PBS containing 0.1% v/v Tween 20 and incubated for 1h at room temperature with goat anti-rabbit horseradish peroxidase-conjugated secondary antibody (ab6721, 1:30000). All antibodies were purchased from Abcam, UK. The immunoreactive proteins were visualized by chemiluminescent method using iBright Western Blot Imaging Systems (Thermo-Fisher Scientific, Germany). Quantitative analysis was performed using iBright Analysis Software (Thermo-Fisher Scientific, Germany). β-actin was used as equal load control.

## **1.14. Statistics**

To investigate the genotype-specific effects on experimental readouts, Student’s t-test for independent samples was used. If experiment included a covariate, one-way ANCOVA test was used with genotype status as independent fixed variable, and sex or age as covariate; if a covariate did not significantly affect the results; it was dropped from the analysis. For the experiments that included drug treatment, a two-way mixed ANCOVA was used with the treatment as the repeated-measurement independent variable, genotype as the fixed independent variable, and sex as the covariate. The same analysis was done for the tests that were performed in many time points, only difference being that time was used as a repeated-measurement independent variable instead of the treatment. In the case of non-normally distributed data, non-parametric alternatives (Mann Witney and Kuskall Wallis tests) were used.

For most of the experiments, no randomization technique was employed in the experimental design due to the nature of independent variable i.e. *CYP2C19* transgenic mice were always compared with *wild type* mice. For the beam-walking experiment which included drug treatment, the *CYP2C19* transgenic and *Wild types* were randomized into treatment groups to ensure that the main readout (beam-crossing time) is equivalent in all experimental groups.

# 2. RESULTS

## **2.1. Tabular representation of genotype specific results of the study**

### **Table S2:** Significance and magnitude of the impact of the genotype on the study results

| Test | Readout | Number of animals  (Outliers; Excluded) | | | %(2C19TG-WT)  [95%CI] | Sign. level |
| --- | --- | --- | --- | --- | --- | --- |
| Rotarod | Latency to fall | | n=97  (O=2) | +0.5%  [-7.1%, +8.2%] | | p=0.90 |
| Beam walking | Beam-crossing time | | n=85  (O=4; E=5*) | +14%  [+6.4%, +22%] | | p=0.0014 |
|  | Number of slips | | n=89  (E=5*) | +457%  [non-parametric] | | p<0.0001 |
| Beam walking  i.p. Saline | Beam-crossing time | | n=89  (O=1, E=3*) | -2.2%  [-10%, +5.5%] | | p=0.81 |
|  | Number of slips | | n=89  (E=4*) | +440% | | p<0.0001 |
| Beam walking  i.p. Raclopride | Beam-crossing time | | n=85  (E=7*) | -0.5%  [-8.8%, +7.8%] | | p=0.81 |
|  | Number of slips | | n=85  (E=7*) | +590% | | p<0.0001 |
| Beam walking  i.p. Ecopipam | Beam-crossing time | | n=83  (E=9*) | -2.6%  [-11%, +6.1%] | | p=0.81 |
|  | Number of slips | | n=83  (E=9*) | +457% | | p<0.0001 |
| Dopamine levels | µl/g of tissue | | n=23 | +15%  [+12%, 19%] | | p<0.0001 |
| **Gd-enhanced neuroimaging** | | | | | | |
| Amygdala volume | Number of voxels | | n=59 | -0.3%  [-2.1, +1.6%] | | p=0.77  q=1.0 |
| Cerebellum volume | Number of voxels | | n=56  (O=3) | -12%  [-15%, -8.9%] | | p<0.0001  q<0.0001 |
| Cerebral Cortex volume | Number of voxels | | n=58  (O=1) | -1.0%  [-2.8%, +0.7%] | | p=0.22  q=1.0 |
| Corpus Striatum volume | Number of voxels | | n=59 | +0.0%  [-1.8%, +1.8%] | | p=0.99  q=1.0 |
| Epithalamus volume | Number of voxels | | n=59 | -4.6%  [-7.4%, -1.8%] | | p=0.0008  q=0.030 |
| Globus Pallidus volume | Number of voxels | | n=59 | -0.8%  [-2.7%, +1.1%] | | p=0.41  q=1.0 |
| Hippocampus volume | Number of voxels | | n=59 | -4.2%  [-6.1%, -2.2%] | | p<0.0001  q=0.0027 |

*continued* **Table S2:** Significance and magnitude of the impact of the genotype on the study results:

| Test | Readout | Number of animals  (Outliers; Excluded) | | | %(2C19TG-WT)  [95%CI] | Sign. level |
| --- | --- | --- | --- | --- | --- | --- |
| Hypothalamus volume | Number of voxels | | n=59 | -0.3%  [-2.2%, +1.6%] | | p=0.79  q=1.0 |
| Inferior Colliculus volume | Number of voxels | | n=59 | -4.2%  [-6.5%, -1.9%] | | p=0.0004  q=0.015 |
| Interpeduncular Nucleus volume | Number of voxels | | n=59 | -2.4%  [-4.5%, -0.3%] | | p=0.030  q=0.89 |
| Nucleus Accumbens volume | Number of voxels | | n=59 | +0.6%  [-1.6%, +2.8%] | | p=0.56  q=1.0 |
| Olfactory Bulb volume | Number of voxels | | n=58  (O=1) | 0.9%  [-1.7%, +3.5%] | | p=0.43  q=1.0 |
| Periaqueductal Gray Matter volume | Number of voxels | | n=54  (O=5) | -2.1%  [-4.2%, -0.1%] | | p=0.40  q=1.0 |
| Pineal Gland volume | Number of voxels | | n=59 | +4.2%  [-3.8%, +12%] | | p=0.29  q=1.0 |
| Pons volume | Number of voxels | | n=59 | -3.9%  [-5.7%, -2.1%] | | p<0.0001  q=0.0017 |
| Septal Nucleus volume | Number of voxels | | n=59 | +0.4%  [-1.9%, +2.7%] | | p=0.76  q=1.0 |
| Substantia Nigra volume | Number of voxels | | n=59 | -3.0%  [-5.0%, -0.9%] | | p=0.006  q=0.18 |
| Superior Colliculus volume | Number of voxels | | n=59 | -1.3%  [-3.3%, +0.6%] | | p=0.18  q=1.0 |
| Thalamus volume | Number of voxels | | n=59 | -2.4%  [-4.4%, -0.4%] | | p=0.022  q=0.68 |
| Unsegmented Midbrain volume | Number of voxels | | n=59 | -3.1%  [-5.0%, -1.2%] | | p=0.0023  q=0.077 |
| **Antioxidant enzyme activity** | | | | | | |
| SOD (hemisphere) | U/g of tissue | | n=58  (O=1, E=5^+^) | +6.0%  [-5.6%, +18%] | | p=0.30  q=1.0 |
| SOD (cerebellum) | U/g of tissue | | n=62  (E=2^+^) | +14%  [+5.8%, +23%] | | p=0.0010  q=0.021 |
| SOD (hippocampus) | U/g of tissue | | n=61  (E=3^+^) | +33%  [+18%, +47%] | | p<0.0001  q=0.0013 |
| Catalase (hemisphere) | U/g of tissue | | n=58  (E=6^+^) | +17%  [-4.6%, +39%] | | p=0.12  q=1.0 |
| Catalase (cerebellum) | U/g of tissue | | n=62  (E=2^+^) | +1.4%  [-13%, +16%] | | p=0.82  q=1.0 |
| Catalase (hippocampus) | U/g of tissue | | n=61  (E=3^+^) | +8.8%  [-4.2%, +21%] | | p=0.43  q=1.0 |
| GPx (hemisphere) | U/g of tissue | | n=58  (E=6^+^) | +38%  [+16%, +59%] | | p=0.0011  q=0.023 |

*continued* **Table S2:** Significance and magnitude of the impact of the genotype on the study results:

| Test | Readout | Number of animals  (Outliers; Excluded) | | | %(2C19TG-WT)  [95%CI] | Sign. level |
| --- | --- | --- | --- | --- | --- | --- |
| GPx (cerebellum) | U/g of tissue | | n=62  (E=2^+^) | +8.8%  [-13%, +30%] | | p=0.16  q=1.0 |
| GPx (hippocampus) | U/g of tissue | | n=61  (E=3^+^) | +8.1%  [-2.9%, +19%] | | p=0.11  q=1.0 |
| GR (hemisphere) | U/g of tissue | | n=53  (O=3, E=8^+^) | +0.0%  [-13%, +13%] | | p=0.91  q=1.0 |
| GR (cerebellum) | U/g of tissue | | n=63  (E=2^+^) | +1.8%  [-9.7%, +13%] | | p=0.78  q=1.0 |
| GR (hippocampus) | U/g of tissue | | n=60  (O=1, E=3) | +23%  [+13%, +35%] | | p<0.0001  q=0.0021 |
| **Antioxidant enzyme expression** | | | | | | |
| SOD1 (hemisphere) | Arbitrary Units | | n=48 | +12%  [-1.4%, +25%] | | p=0.084  q=1.0 |
| SOD1 (cerebellum) | Arbitrary Units | | n=41  (O=7) | +10%  [-5.3%, +26%] | | p=0.26  q=1.0 |
| SOD1 (hippocampus) | Arbitrary Units | | n=48 | +5.2%  [-11%, +16%] | | p=0.52  q=1.0 |
| SOD2 (hemisphere) | Arbitrary Units | | n=47  (O=1) | +5.7%  [-4.7%, +16%] | | p=0.28  q=1.0 |
| SOD2 (cerebellum) | Arbitrary Units | | n=44  (O=2, E=2^+^) | +23%  [+7.9%, +39%] | | p=0.0074  q=0.21 |
| SOD2 (hippocampus) | Arbitrary Units | | n=46  (O=2) | -4.3%  [-14%, +4.9%] | | p=0.36  q=1.0 |
| Catalase (hemisphere) | Arbitrary Units | | n=43  (O=5) | -0.2%  [-7.2%, +6.8%] | | p=0.98  q=1.0 |
| Catalase (cerebellum) | Arbitrary Units | | n=46  (E=2^+^) | +6.5%  [-6.2%, +19%] | | p=0.32  q=1.0 |
| Catalase (hippocampus) | Arbitrary Units | | n=35  (O=1, E=12^+^) | -0.1%  [-18%, +18%] | | p=0.98  q=0.98 |
| GPx (hemisphere) | Arbitrary Units | | n=48 | +3.8%  [-9.8%, +17%] | | p=0.58  q=1.0 |
| GPx (cerebellum) | Arbitrary Units | | n=46  (O=2) | -3.5%  [-12%, +5.2%] | | p=0.44  q=1.0 |
| GPx (hippocampus) | Arbitrary Units | | n=48 | +6.7%  [-5.2%, +19%] | | p=0.27  q=1.0 |
| GR (hemisphere) | Arbitrary Units | | n=48 | -5.4%  [-15%, +4.2%] | | p=0.28  q=1.0 |
| GR (cerebellum) | Arbitrary Units | | n=48 | +4.7%  [-9.7%, +16%] | | p=0.52  q=1.0 |
| GR (hippocampus) | Arbitrary Units | | n=47  (O=1) | +1.6%  [-9.4%, +13%] | | p=0.79  q=1.0 |

*continued* **Table S2:** Significance and magnitude of the impact of the genotype on the study results:

| Test | Readout | Number of animals  (Outliers; Excluded) | | | %(2C19TG-WT)  [95%CI] | Sign. level |
| --- | --- | --- | --- | --- | --- | --- |
| **Number of midbrain dopaminergic neurons** | | | | | | |
| Coronal section  -2.80mm from Bregma | Substantia Nigra | | n=18 | +1.3%  [-12.9%, +15.5%] | | p=0.86  q=1.0 |
|  | Ventral Tegmental Area | | n=18 | +8.3%  [-10%, +27%] | | p=0.36  q=1.0 |
| Coronal section  -2.92mm from Bregma | Substantia Nigra | | n=18 | -10.5%  [-22.4%, +1.4%] | | p=0.11  q=1.0 |
|  | Ventral Tegmental Area | | n=18 | -2.4%  [-17%, -13%] | | p=0.77  q=1.0 |
| Coronal section  -3.08mm from Bregma | Substantia Nigra | | n=18 | -4.9%  [-24.7%, +14.9%] | | p=0.65 q=1.0 |
|  | Ventral Tegmental Area | | n=18 | 2.3%  [-13%, -18%] | | p=0.75  q=1.0 |
| Coronal section  -3.16mm from Bregma | Substantia Nigra | | n=18 | -3.5%  [-23.3%, +16.4%] | | p=0.69  q=1.0 |
|  | Ventral Tegmental Area | | n=18 | -2.6%  [-28%, +22%] | | p=0.83  q=1.0 |
| Coronal section  -3.28mm from Bregma | Substantia Nigra | | n=18 | -12.2%  [-34.4%, +9.9%] | | p=0.19  q=1.0 |
|  | Ventral Tegmental Area | | n=18 | -4.4%  [-29%, +20%] | | p=0.56  q=1.0 |
| Coronal section  -3.40mm from Bregma | Substantia Nigra | | n=18 | -0.6%  [-27.8%, +26.6%] | | p=0.96  q=0.96 |
|  | Ventral Tegmental Area | | n=18 | +1.3%  [%-21, +8.1%] | | p=0.92  q=0.92 |
| Coronal section  -3.52mm from Bregma | Substantia Nigra | | n=18 | -15.0%  [-27.8%, -2.1%] | | p=0.037  q=0.52 |
|  | Ventral Tegmental Area | | n=18 | -6.7%  [-21%, +8.1%] | | p=0.39  q=1.0 |
| Coronal section  -3.64mm from Bregma | Substantia Nigra | | n=18 | -1.7%  [-22.1%, 18.6%] | | p=0.83  q=1.0 |
|  | Ventral Tegmental Area | | n=18 | -6.4%  [-32%, +19%] | | p=0.63  q=1.0 |
| Coronal section  -3.80mm from Bregma | Substantia Nigra | | n=17  (O=1) | -6.9%  [-20.1%, +6.3%] | | p=0.15  q=1.0 |
|  | Ventral Tegmental Area | | n=17  (O=1) | +1.7%  [-14%, +18%] | | p=0.83  q=1.0 |

*continued* **Table S2:** Significance and magnitude of the impact of the genotype on the study results:

| Test | Readout | Number of animals  (Outliers; Excluded) | | | %(2C19TG-WT)  [95%CI] | Sign. level |
| --- | --- | --- | --- | --- | --- | --- |
| **Gait analysis** | | | | | | |
| Body weight measurement | Postnatal day 21 | | n=53 | -13.5%  [-19.3%, -7.7%] | | p<0.0001 |
|  | Postnatal day 28 | | n=53 | -6.9%  [-11.7%, -2.1%] | | p=0.0054 |
|  | Postnatal day 35 | | n=53 | -1.6%  [-5.3%, +2.1%] | | p=0.39 |
|  | Postnatal day 41 | | n=53 | -0.9%  [-4.5%, +2.7%] | | p=0.62 |
| Maximal hindpaw height | Postnatal week 5 | | n=53 | +114%  [+102%, +125%] | | p<0.0001 |
|  | Postnatal week 6 | | n=53 | +103%  [+90%, +116%] | | p<0.0001 |
|  | Postnatal week 7 | | n=53 | +97%  [+85%, +108%] | | p<0.0001 |
|  | Postnatal week 8 | | n=53 | +92%  [+80%, +104%] | | p<0.0001 |
|  | Postnatal week >30 | | n=30 | +95%  [+78%, +112%] | | p<0.0001 |
| Footprint test:  Left forepaw stride length | Young mice  (5-8 weeks) | | n=42  E=10* | +0.2%  [-3.6%, +4.1%] | | p=0.91 |
|  | Adult mice  (>30 weeks) | | n=30 | -2.6%  [-11%, +6.1%] | | p=0.55 |
| Footprint test:  Right forepaw stride length | Young mice  (5-8 weeks) | | n=42  E=10* | +0.9%  [-2.9%, +4.6%] | | p=0.64 |
|  | Adult mice  (>30 weeks) | | n=30 | -2.2%  [-11%, +6.3%] | | p=0.60 |
| Footprint test:  Left hindpaw stride length | Young mice  (5-8 weeks) | | n=42  E=10* | +0.2%  [-3.8%, +4.1%] | | p=0.93 |
|  | Adult mice  (>30 weeks) | | n=30 | -1.1%  [-10.5%, +8.2%] | | p=0.80 |
| Footprint test:  Right hindpaw stride length | Young mice  (5-8 weeks) | | n=42  E=10* | +0.8%  [-3.1%, +4.7%] | | p=0.70 |
|  | Adult mice  (>30 weeks) | | n=30 | -0.5%  [-9.5%, +8.6%] | | p=0.92 |
| Footprint test:  Front base width | Young mice  (5-8 weeks) | | n=41  O=1, E=10* | -1.3%  [-4.2%, +7.0%] | | p=0.62 |
|  | Adult mice  (>30 weeks) | | n=30 | -2.3%  [-13%, +8.5%] | | p=0.66 |
| Footprint test:  Hind base width | Young mice  (5-8 weeks) | | n=42  E=10* | -3.0%  [-7.1%, +1.1%] | | p=0.15 |
|  | Adult mice  (>30 weeks) | | n=30 | -2.9%  [-12%, +6.1%] | | p=0.51 |

*continued* **Table S2:** Significance and magnitude of the impact of the genotype on the study results:

| Test | Readout | Number of animals  (Outliers; Excluded) | | | %(2C19TG-WT)  [95%CI] | Sign. level |
| --- | --- | --- | --- | --- | --- | --- |
| **Gait analysis** | | | | | | |
| Footprint test:  Left overlap | Young mice  (5-8 weeks) | | n=40  O=2, E=10* | +6.8%  [-8.9%, +22%] | | p=0.39 |
|  | Adult mice  (>30 weeks) | | n=30 | 5.5%  [-20%, +31%] | | p=0.66 |
| Footprint test:  Right overlap | Young mice  (5-8 weeks) | | n=41  O=1, E=10* | +10.3%  [-4.4%, +25%] | | p=0.16 |
|  | Adult mice  (>30 weeks) | | n=30 | -8.3%  [-40%, +23%] | | p=0.60 |

*Excluded due to failed test.

^+^Excluded due to invalid measurement.

## **2.2. Effects of covariates**

Statistical significance of the effect of covariates on the results is presented in the TableS3 and TableS4:

### **Table S3:** Significance of the impact of sex as a covariate in the study results

| Test | *Readout* | Total number of animals | Significance |
| --- | --- | --- | --- |
| Rotarod | Latency to fall | n=97 | p=0.0001 |
| Beam walking | Beam-Crossing time | n=85 | p=0.70 |
|  | Number of slips |  | p=0.26 |
| Beam walking – i.p. Saline | Beam-Crossing time | n=85 | p=0.11 |
|  | Number of slips | n=89 | p=0.79 |
| Beam walking – i.p. Raclopride | Beam-Crossing time | n=85 | p=0.030 |
|  | Number of slips |  | p=0.44 |
| Beam walking – i.p. Ecopipam | Beam-Crossing time | n=83 | p=0.0008 |
|  | Number of slips |  | p=0.58 |
| Dopamine levels | µl/g of tissue | n=23 | N/A |
| Gd-enhanced neuroimaging | | | |
| Amygdala volume | Number of voxels | n=59 | p=0.44  q=1.0 |
| Cerebellum volume | Number of voxels | n=56 | p=0.030  q=0.87 |
| Cerebral Cortex volume | Number of voxels | n=58 | p=0.070  q=1.0 |
| Corpus Striatum volume | Number of voxels | n=59 | p=0.27  q=1.0 |
| Epithalamus volume | Number of voxels | n=59 | p=0.010  q=0.33 |
| Globus Pallidus volume | Number of voxels | n=59 | p=0.72  q=1.0 |
| Hippocampus volume | Number of voxels | n=59 | p=0.58  q=1.00 |
| Hypothalamus volume | Number of voxels | n=59 | p=0.19  q=1.0 |
| Inferior Colliculus volume | Number of voxels | n=59 | p=0.047  q=1.0 |
| Interpeduncular Nucleus volume | Number of voxels | n=59 | p=0.41  q=1.0 |
| Nucleus Accumbens volume | Number of voxels | n=59 | p=0.35  q=1.0 |
| Olfactory Bulb volume | Number of voxels | n=58 | p=0.0022  q=0.078 |

*continued* **Table S3**: Significance of the impact of sex as a covariate in the study results

| Test | *Readout* | Total number of animals | Significance |
| --- | --- | --- | --- |
| Periaqueductal Gray Matter volume | Number of voxels | n=54 | p=0.51  q=1.0 |
| Pineal Gland volume | Number of voxels | n=59 | p=0.32  q=1.0 |
| Pons volume | Number of voxels | n=59 | p=0.13  q=1.0 |
| Septal Nucleus volume | Number of voxels | n=59 | p=0.82  q=1.0 |
| Substantia Nigra volume | Number of voxels | n=59 | p=0.41  q=1.0 |
| Superior Colliculus volume | Number of voxels | n=59 | p=0.57  q=1.0 |
| Thalamus volume | Number of voxels | n=59 | p=0.12  q=1.0 |
| Unsegmented Midbrain volume | Number of voxels | n=59 | p=0.70  q=1.0 |
| Antioxidant enzyme activity | | | |
| SOD (hemisphere) | U/g of tissue | n=58 | p=0.35  q=1.0 |
| SOD (cerebellum) | U/g of tissue | n=62 | p=0.030  q=0.58 |
| SOD (hippocampus) | U/g of tissue | n=61 | p=0.70  q=1.0 |
| Catalase (hemisphere) | U/g of tissue | n=58 | p=0.15  q=1.0 |
| Catalase (cerebellum) | U/g of tissue | n=62 | p=0.13  q=1.0 |
| Catalase (hippocampus) | U/g of tissue | n=61 | p=0.89  q=1.0 |
| GPx (hemisphere) | U/g of tissue | n=58 | p=0.18  q=1.0 |
| GPx (cerebellum) | U/g of tissue | n=62 | p=0.078  q=1.0 |
| GPx (hippocampus) | U/g of tissue | n=61 | p=0.0014  q=0.029 |
| GR (hemisphere) | U/g of tissue | n=53 | p=0.069  q=1.0 |
| GR (cerebellum) | U/g of tissue | n=63 | p=0.26  q=1.0 |
| GR (hippocampus) | U/g of tissue | n=60 | p=0.12  q=1.0 |

*continued* **Table S3:** Significance of the impact of sex as a covariate in the study results

| Test | *Readout* | Total number of animals | Significance |
| --- | --- | --- | --- |
| Antioxidative enzyme expression | | | |
| SOD1 (hemisphere) | Arbitrary Units | n=48 | p=0.23  q=1.0 |
| SOD1 (cerebellum) | Arbitrary Units | n=41 | p=0.45  q=1.0 |
| SOD1 (hippocampus) | Arbitrary Units | n=48 | p=0.31  q=1.0 |
| SOD2 (hemisphere) | Arbitrary Units | n=47 | p=0.0039  q=0.12 |
| SOD2 (cerebellum) | Arbitrary Units | n=44 | p=0.95  q=0.95 |
| SOD2 (hippocampus) | Arbitrary Units | n=46 | p=0.68  q=1.0 |
| Catalase (hemisphere) | Arbitrary Units | n=43 | p=0.59  q=1.0 |
| Catalase (cerebellum) | Arbitrary Units | n=46 | p=0.50  q=1.0 |
| Catalase (hippocampus) | Arbitrary Units | n=35 | p=0.49  q=1.0 |
| GPx (hemisphere) | Arbitrary Units | n=48 | p=0.82  q=1.0 |
| GPx (cerebellum) | Arbitrary Units | n=46 | p=0.78  q=1.0 |
| GPx (hippocampus) | Arbitrary Units | n=48 | p=0.18  q=1.0 |
| GR (hemisphere) | Arbitrary Units | n=48 | p=0.61  q=1.0 |
| GR (cerebellum) | Arbitrary Units | n=48 | p=0.67  q=1.0 |
| GR (hippocampus) | Arbitrary Units | n=47 | p=0.35  q=1.0 |

*continued* **Table S3:** Significance of the impact of sex as a covariate in the study results

| Test | *Readout* | Total number of animals | Significance |
| --- | --- | --- | --- |
| Gait analysis | | | |
| Body weight measurement | Postnatal day 21 | n=53 | p<0.0001 |
|  | Postnatal day 28 | n=53 | p<0.0001 |
|  | Postnatal day 35 | n=53 | p<0.0001 |
|  | Postnatal day 41 | n=53 | p<0.0001 |
| Maximal hindpaw height | Postnatal week 5 | n=53 | p=0.87 |
|  | Postnatal week 6 | n=53 | p=0.15 |
|  | Postnatal week 7 | n=53 | p=0.069 |
|  | Postnatal week 8 | n=53 | p=0.055 |
|  | Postnatal week >30 | n=30 | p=0.16 |
| Footprint test:  Left forepaw stride length | Young mice  (5-8 weeks) | n=42 | p=0.0042 |
|  | Adult mice (>30 weeks) | n=30 | p=0.61 |
| Footprint test:  Right forepaw stride length | Young mice  (5-8 weeks) | n=42 | p=0.025 |
|  | Adult mice (>30 weeks) | n=30 | p=0.80 |
| Footprint test:  Left hindpaw stride length | Young mice  (5-8 weeks) | n=42 | p=0.0022 |
|  | Adult mice (>30 weeks) | n=30 | p=0.47 |
| Footprint test:  Right hindpaw stride length | Young mice  (5-8 weeks) | n=42 | p=0.026 |
|  | Adult mice (>30 weeks) | n=30 | p=0.60 |
| Footprint test:  Front base width | Young mice  (5-8 weeks) | n=41 | p=0.32 |
|  | Adult mice (>30 weeks) | n=30 | p=0.25 |
| Footprint test:  Hind base width | Young mice  (5-8 weeks) | n=42 | p=0.10 |
|  | Adult mice (>30 weeks) | n=30 | p=0.85 |
| Footprint test:  Left overlap | Young mice  (5-8 weeks) | n=40 | p=0.018 |
|  | Adult mice (>30 weeks) | n=30 | p=0.65 |
| Footprint test:  Right overlap | Young mice  (5-8 weeks) | n=41 | p=0.0047 |
|  | Adult mice (>30 weeks) | n=30 | p=0.33 |

### **Table S4:** Significance of the impact of age as a covariate in the study results

| Test | *Readout* | Total number of animals | Significance |
| --- | --- | --- | --- |
| Coronal section  -2.80mm from Bregma | Number of neurons in  Substantia Nigra | n=18 | p=0.58  q=1.0 |
|  | Number of neurons in  Ventral Tegmental Area | n=18 | p=0.079  q=1.00 |
| Coronal section  -2.92mm from Bregma | Number of neurons in  Substantia Nigra | n=18 | p=0.83  q=1.0 |
|  | Number of neurons in  Ventral Tegmental Area | n=18 | p=0.70  q=1.0 |
| Coronal section  -3.08mm from Bregma | Number of neurons in  Substantia Nigra | n=18 | p=0.94  q=1.0 |
|  | Number of neurons in  Ventral Tegmental Area | n=18 | p=0.044  q=0.75 |
| Coronal section  -3.16mm from Bregma | Number of neurons in  Substantia Nigra | n=18 | p=0.014  q=0.21 |
|  | Number of neurons in  Ventral Tegmental Area | n=18 | p=0.18  q=1.00 |
| Coronal section  -3.28mm from Bregma | Number of neurons in  Substantia Nigra | n=18 | p=0.005  q=0.09 |
|  | Number of neurons in  Ventral Tegmental Area | n=18 | p<0.0001  q=0.0012 |
| Coronal section  -3.40mm from Bregma | Number of neurons in  Substantia Nigra | n=18 | p=0.006  q=0.102 |
|  | Number of neurons in  Ventral Tegmental Area | n=18 | p=0.52  q=1.0 |
| Coronal section  -3.52mm from Bregma | Number of neurons in  Substantia Nigra | n=18 | p=0.33  q=1.0 |
|  | Number of neurons in  Ventral Tegmental Area | n=18 | p=0.28  q=1.0 |
| Coronal section  -3.64mm from Bregma | Number of neurons in  Substantia Nigra | n=18 | p=0.006  q=0.096 |
|  | Number of neurons in  Ventral Tegmental Area | n=18 | p=0.39  q=1.0 |
| Coronal section  -3.80mm from Bregma | Number of neurons in  Substantia Nigra | n=17 | p=0.11  q=1.00 |
|  | Number of neurons in  Ventral Tegmental Area | n=17 | p=0.12  q=1.00 |

**2.3. Determination of the cohort size**

One mouse was considered as a single experimental unit. Three separate mice cohorts, each consisting out of one experimental (*CYP2C19* transgenic mice - TG) and one control (*wild type* - WT) group, were used for this study:

- **Cohort 1:** For the dopamine concentration and dopamine metabolite concentration measurements in various brain regions.
  - n=23; 13 WT + 11 TG; all males; 3 months old mice
  - n=20; 7 WT + 13 TG; embryos at embryotic day E18.5
  - Procedure: Sacrificed for the sampling of the entire brain
- **Cohort 2:** For the immunohistochemistry and neuromelanin staining
  - n=18; 4 WT + 4 TG (15-month-old); 5 TG + 5 WT (6-month-old); all males
  - Procedure: Sacrificed for the production of the histological brain microscope slides
- **Cohort 3:** For the purpose of the motoric behavior tests, neuroimaging, and antioxidative enzyme status
  - n= 99; 47WT + 52 TG; 3-6 months old; males + females
  - Procedure: First, rotarod test was performed followed with baseline beam-walking test.
  - Then, beam-walking test was repeated under antidopaminergic treatment protocol described previously.
  - Then, 60 out of 99 animals were sacrificed for the purpose of postmortem neuroimaging.
  - Remaining 39 animal cohort was expanded with 25 more animals (12 WT + 13 TG, 3-6 months old), and this groups of 64 animals was sacrificed for the sampling of the 3 brain regions for the measurement of the antioxidative enzyme expression and activity.
- **Cohort 4:** For the Post hoc gait analysis
  - N=53; 26WT+27TG; 3 weeks old
  - Procedure: Body weight was measured from postnatal week 3 until week 8,
  - Footprint test and hindpaw height measurement were performed in weeks 5-8.
  - Animals are returned in the colony for future experiments or colony expansion.

Number of animals for the cohort 3, used for the majority of the experiments, was decided based on the standard formula^10^:

n = $\frac{{(Z_{\alpha/2}+Z_{\beta})}^{2}\times2\times\sigma^{2}}{d^{2}}$

where the target difference between the groups (*d*) was set at 10%, since *CYP2C19* transgenic mice were expected to show mild motoric impairment of around 10% in the rotarod and beam-walking test, if any. Also, standard deviation (σ) of 20% was chosen for the calculations based on the result of the pilot study^11^, confidence level was set at 95% and power to 80%. This analysis predicted that 126 animals (63 TG + 63 WT) were needed for the experiment with the given parameters, but this number was limited to 100 animals due to practical reasons. Since large animal cohort was needed, special care was taken to re-use the same cohort in as many experiments in order to reduce animal suffering as much as possible.

Other cohorts were selected in a way to produce most meaningful results with as few animals as possible.

**2.4. Bilaterality of abnormal hindpaw elevation in *CYP2C19* transgenic mice**

### **Table S5:** Frequencies of TG animals with unilateral or bilateral motoric phenotypes

| Time point | Ratio of animals with unilateral phenotype | Ratio of animals with bilateral phenotype |
| --- | --- | --- |
| Postnatal week 5 | 0/53 | 53/53 |
| Postnatal week 6 | 2/53 | 51/53 |
| Postnatal week 7 | 4/53 | 49/53 |
| Postnatal week 8 | 5/53 | 48/53 |
| Postnatal week >30 | 28/30 | 2/30 |

## **2.5. Stride lengths in young and adult *CYP2C19* transgenic (TG) and *Wild type* (WT) mice**


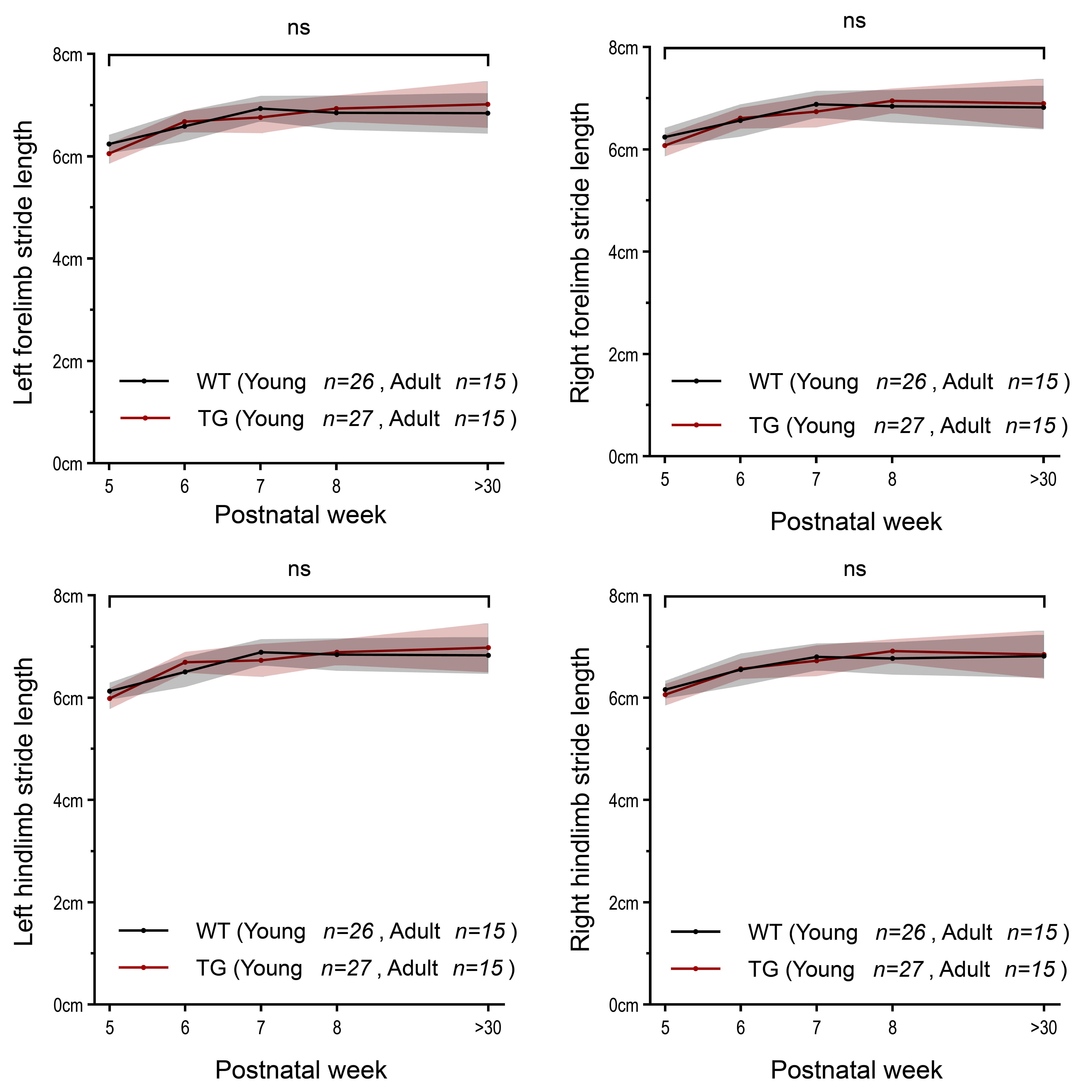


### **Figure S2:** Footprint analysis: Stride lengths for every paw in young and adult mice.

Step length gradually increased with time in all young animals. No significant changes between the test groups were observed in the length of any paw at any time point.

## **2.6. Expression of the anti-oxidative enzymes in the brain tissue of *CYP2C19* transgenic mice**


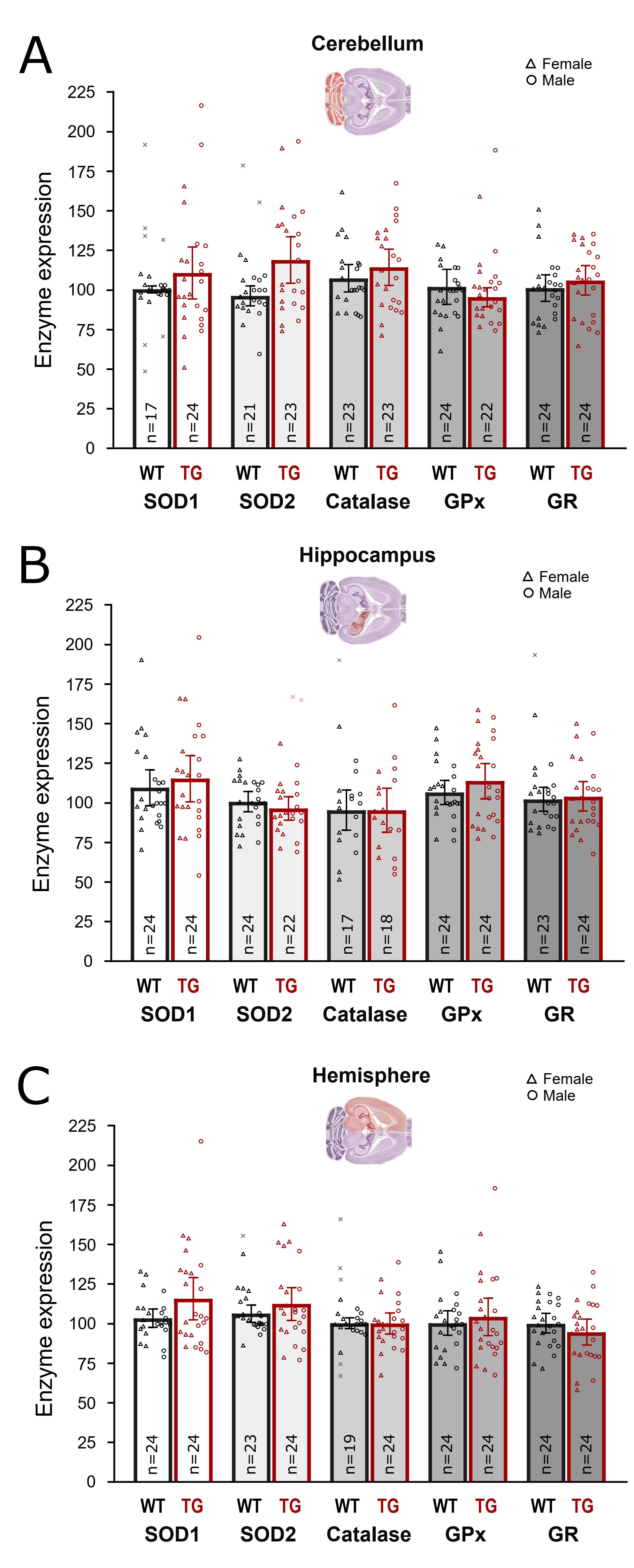


### **Figure S3:** Expression of antioxidant enzymes in 3 brain regions.

There was no change between *CYP2C19* transgenic (TG) and w*ild type* (WT) mice in the expression of either **(A)** Cupper-Zink Superoxide dismutase (SOD1), **(B)** Manganese Superoxide dismutase (SOD2), **(C)** Catalase **(D)** Glutathione peroxidase (GPx) and **(E)** Glutathione reductase (GR) in whole hemisphere, cerebellum or hippocampus. Primary analysis showed significant increase in the expression of SOD2 enzyme in cerebellum (23% increase 95%CI: [7.9%, 39%], p=0.0074) in TG mice but this change did not remain significant after correction for multiple comparisons (q=0.12). Enzyme relative concentrations were determined by western blot method and concentrations were expressed in arbitrary units.

# 3. REFERENCES

1. Lalonde R, Strazielle C. Brain regions and genes affecting limb-clasping responses. Brain Res Rev 2011;67(1-2):252-259.
2. Brooks SP, Dunnett SB. Tests to assess motor phenotype in mice: a user's guide. Nat Rev Neurosci 2009;10(7):519-529.
3. Najmanová V, Rambousek L, Syslová K, et al. LC-ESI-MS-MS Method for Monitoring Dopamine, Serotonin and Their Metabolites in Brain Tissue. Chromatographia 2011;73(1):143-149.
4. Paxinos G, Franklin KB. Paxinos and Franklin's the mouse brain in stereotaxic coordinates. Academic press 2019.
5. Lowry OH, Rosebrough NJ, Farr AL, Randall RJ. Protein measurement with the Folin phenol reagent. J Biol Chem 1951;193(1):265-275.
6. Misra HP, Fridovich I. The generation of superoxide radical during the autoxidation of hemoglobin. J Biol Chem 1972;247(21):6960-6962.
7. Beutler E. Catalase: A Manual of biochemical methods. In: Beutler E. (Ed.) Red Cell Metabolism, New York: Grune and Stratton. 1982:105–106.
8. Paglia DE, Valentine WN. Studies on the quantitative and qualitative characterization of erythrocyte glutathione peroxidase. J Lab Clin Med 1967;70(1):158-169.
9. Glatzle D, Vuilleumier JP, Weber F, Decker K. Glutathione reductase test with whole blood, a convenient procedure for the assessment of the riboflavin status in humans. Experientia 1974;30(6):665-667.
10. Estimation of Sample Size and Power for Comparing Two Means section in Rosner, B., (2015). Fundamentals of Biostatistics. 8th ed. USA: Cengage Learning
11. Milosavljević F, Vučić M, Manojlović M, et al. P.2.25 Transgenic mouse, carrier of human *CYP2C19* gene, as an animal model for hyperdopaminergism-induced hyperkinesia. European Neuropsychopharmacology 2019;29:S672-S673.
